# A test for meiotic drive in hybrids between Australian and Timor zebra finches

**DOI:** 10.1002/ece3.6951

**Published:** 2020-11-03

**Authors:** Ulrich Knief, Wolfgang Forstmeier, Yifan Pei, Jochen Wolf, Bart Kempenaers

**Affiliations:** ^1^ Department of Behavioural Ecology and Evolutionary Genetics Max Planck Institute for Ornithology Seewiesen Germany; ^2^ Division of Evolutionary Biology Faculty of Biology Ludwig Maximilian University of Munich Planegg‐Martinsried Germany

**Keywords:** centromere drive, cryptic drive, meiotic drive, telomere drive, transmission distortion

## Abstract

Meiotic drivers have been proposed as a potent evolutionary force underlying genetic and phenotypic variation, genome structure, and also speciation. Due to their strong selective advantage, they are expected to rapidly spread through a population despite potentially detrimental effects on organismal fitness. Once fixed, autosomal drivers are cryptic within populations and only become visible in between‐population crosses lacking the driver or corresponding suppressor. However, the assumed ubiquity of meiotic drivers has rarely been assessed in crosses between populations or species. Here we test for meiotic drive in hybrid embryos and offspring of Timor and Australian zebra finches—subspecies that have evolved in isolation for about two million years—using 38,541 informative transmissions of 56 markers linked to either centromeres or distal chromosome ends. We did not find evidence for meiotic driver loci on specific chromosomes. However, we observed a weak overall transmission bias toward Timor alleles at centromeres in females (transmission probability of Australian alleles of 47%, nominal *p* = 6 × 10^–5^). While this is in line with the centromere drive theory, it goes against the expectation that the subspecies with the larger effective population size (i.e., the Australian zebra finch) should have evolved the more potent meiotic drivers. We thus caution against interpreting our finding as definite evidence for centromeric drive. Yet, weak centromeric meiotic drivers may be more common than generally anticipated and we encourage further studies that are designed to detect also small effect meiotic drivers.

## Graphical abstract

We test for meiotic drive in hybrid embryos and offspring of Timor and Australian zebra finches. We did not find evidence for meiotic driver loci on specific chromosomes but observed an overall transmission bias towards Timor alleles at centromeres in females.

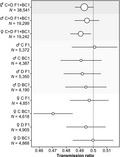

## 1 INTRODUCTION

Among the few universally accepted laws in biology are the principles of Mendelian inheritance, which set the theoretical framework for the transmission of genetic material in sexually reproducing organisms from one generation to the next (Mendel, 1865). One of Mendel's central observations was that the two alleles at each locus of a diploid organism are transmitted to the next generation with equal probability. However, rules of general relevance in biology often come with exceptions; indeed, deviations from fair Mendelian segregation have been observed in a range of organisms (Lindholm et al., 2016). Alleles that lead to deviations from Mendelian segregation are collectively referred to as meiotic drivers (Lindholm et al., 2016). Here, we define them as entire chromatids, chromosomes, or parts thereof, which outcompete their homologs (or corresponding sex chromosome) *within* a parental individual over access to the next generation. Meiotic drivers are often difficult to distinguish from other pre‐ or postzygotic processes that might appear to bias fair Mendelian segregation. These usually result from interactions *between* parental genotypes, such as a genetic conflict among parental individuals (e.g., female control over paternity) or early viability selection (summarized in Knief, Schielzeth, et al., 2015).

Whether meiotic drive can be observed typically depends on the fitness consequences of the driver. If a meiotic driver has no detrimental effects on the fitness of the carrier, the drive allele is expected to spread rapidly to fixation within a population (Traulsen & Reed, 2012). The distortion caused by such a drive allele will then no longer be visible because the nondriving ancestral allele has been driven to extinction. Given the short evolutionary trajectory of such past events, most of the known drivers impose a major cost on individual fitness which prevents rapid elimination of the competing ancestral allele (Burt & Trivers, 2006). For instance, drive alleles are often linked to recessive deleterious mutations causing the death of homozygous offspring in heterozygote–heterozygote pairings (Fishman & Kelly, 2015), leading to retention of the ancestral allele. Some drivers also reduce the number of gametes produced by heterozygous carriers (Sutter & Lindholm, 2015), such that a disadvantage in sperm competition further slows down the spread of the drive allele. The resulting decline in fitness may then favor the evolution of unlinked suppressors of drive, which might be able to restore fair Mendelian segregation, making the previous distortion again invisible (“cryptic drive system”; Frank, 1991; Hurst & Pomiankowski, 1991; Sandler & Novitski, 1957). Overall, meiotic drivers might thus only be transiently active within a population (Meyer et al., 2012).

Cryptic meiotic drivers can be detected by crossing individuals from diverged populations. If a meiotic driver evolved in one, but not in the other population, the driver will be reestablished in the naïve genetic background of hybrid individuals (Fishman & Willis, 2005; Hurst & Werren, 2001). A complicating factor is that other processes may also lead to apparent deviations from fair Mendelian segregation in these crosses. For example, Bateson–Dobzhansky–Muller (BDM) incompatibilities can arise when two or more genes independently accumulate mutations that spread to fixation in isolated populations. Although these mutated genes function perfectly within the population they first occurred in, they might be malfunctioning in hybrids because of epistatic interactions (Bateson, 1909; Dobzhansky, 1937; Muller, 1942). If these detrimental interactions are additive or dominant, their effects are observable in the F1 hybrids. If they are recessive, these effects will only appear in the second generation after hybridization, not in the F1 hybrids themselves. The heterogametic sex (in birds the female, being ZW) is usually more affected (Haldane's rule; Haldane, 1922), which may lead to biased sex ratios. Importantly, any such detrimental effect on gamete or offspring viability will result in apparent deviations from Mendelian inheritance when considering only surviving offspring. Hence, to distinguish meiotic drive from BDM incompatibilities, it is essential to monitor the genotypes of all offspring, including embryos that failed to develop, and also sample apparently infertile eggs.

To distinguish between meiotic drivers and other processes that bias transmission ratios, we can make use of the fact that meiotic drivers generally act in a sex‐specific manner whereas most of the other processes take place in both sexes (Lindholm et al., 2016). In females, a single oogonium leads to the formation of one oocyte and three polar bodies. Because the latter represent an evolutionary dead end, a meiotic driver may act by outcompeting its homologous chromosome for inclusion into the oocyte (Axelrod & Hamilton, 1981; Pardo‐Manuel de Villena & Sapienza, 2001). Following Sandler and Novitski (1957), we call this “chromosomal meiotic drive.” In contrast, male meiosis is symmetric, because a single primary spermatocyte gives rise to four functional spermatozoa. Consequently, meiotic drivers in males must act postmeiotically, causing disruption or out‐performance of those sperm that do not carry the driving allele (“genic meiotic drive”; Pardo‐Manuel de Villena & Sapienza, 2001; Sandler & Novitski, 1957).

Genic meiotic drivers depend on specific genes (a drive and a target locus) that are often linked by an inversion and can be localized anywhere in the genome. Well‐known examples include the *t*‐complex in mice (*Mus musculus*; reviewed in Lyttle, 1991), the *SD* locus in *Drosophila melanogaster* (reviewed in Lyttle, 1991), the *wtf* genes in the yeast *Schizosaccharomyces pombe* (Eickbush et al., 2019), and the recently discovered *ORF*‐system in rice (*Oryza meridionalis*; Yu et al., 2018). These systems involve “poison” and “antidote” alleles that first disable all sperm or pollen and subsequently resurrect only those of their own genotype.

In contrast, chromosomal meiotic drive arises as a consequence of specific chromosomal structural elements. During meiosis, the spindle apparatus attaches to all chromosomes at their centromeres and separates the homologous chromosomes (meiosis I) and subsequently sister chromatids (meiosis II) into daughter cells. Provided that the spindle apparatus exhibits a functional polarity that differentiates the oocyte from the polar bodies, length and sequence polymorphisms at or near the centromere might enable some centromeres to preferentially attach to the spindle leading to the oocyte (Pardo‐Manuel de Villena & Sapienza, 2001). In birds, the oocyte spindle in meiosis I is positioned close to the egg cortex, perpendicular to the egg surface. The first polar body forms toward the cortical side (Yoshimura et al., 1993), such that the distance to the egg cortex provides spatial information for a driving chromosome (Rutkowska & Badyaev, 2008). Recently, it was shown in mice that centromeres with more minor satellite repeats attract more spindle microtubules (Chmátal et al., 2014; Iwata‐Otsubo et al., 2017). It was further shown that egg and polar body spindle are differentially tyrosinated as a result of their distance to the egg cortex, which allows those centromeres with more minor satellite repeats to preferentially attach to the egg spindle (Akera et al., 2017). As a result, chromosomes with more repeats were transmitted to around 62%–81% of the oocytes (Akera et al., 2019; Iwata‐Otsubo et al., 2017), confirming the idea of female chromosomal meiotic drive at centromeres (Henikoff et al., 2001). Interestingly, the mice strains used by Iwata‐Otsubo et al. (2017) and Akera et al. (2019) differed consistently in their number of minor satellite repeats at centromeres across all chromosomes, resulting in genome‐wide meiotic drive.

Besides meiotic drive that acts on the centromeres, some organisms like maize (Burt & Trivers, 2006) have also evolved “neocentromeres” that are typically located about 50 cM away from the centromere. Their driving mechanism requires a single crossover between the centromere and the driving neocentromere (Dawe & Hiatt, 2004), such that each chromosome contains one neocentromere in meiosis I. Neocentromeres are then pulled toward the spindle poles ahead of the centromeres by a specialized kinesin (Dawe et al., 2018), such that the neocentromeres end up in the outer cells of a linear tetrad and one of them forms the egg cell (Rhoades, 1952). The kinesin gene is tightly linked to the neocentromere, and both are passed on together (Dawe et al., 2018). Hence, drive may also be expected at sites that are ~50 cM away from the centromere.

Birds are an ideal model to study meiotic drive directly, because all embryos resulting from a specific pairing, that is, every egg that a female produces, can be sampled and investigated, including infertile eggs. Despite this, tests for drive have been conducted in only two bird species so far. Intriguingly, both studies suggested meiotic drive systems (chicken [*Gallus gallus*]: Axelsson et al., 2010; Australian zebra finch [*Taeniopygia guttata castanotis*]: Knief, Schielzeth, et al., 2015). Axelsson et al. (2010) described a chromosomal meiotic driver at the centromere of chicken chromosome *Gga1*, for which also a centromeric length polymorphism exists (Shang et al., 2010). In Australian zebra finches, the driver was located on chromosome *Tgu2* and acted in both sexes (Knief, Schielzeth, et al., 2015), but the molecular mechanism requires further study. Many (avian) species show a drastic drop in nucleotide diversity toward putative centromeric regions of almost all chromosomes (Burri et al., 2015; Delmore et al., 2015; Ellegren et al., 2012; Irwin et al., 2016; Knief & Forstmeier, 2016; Knief et al., 2016; Laine et al., 2016; Van Doren et al., 2017; Vijay et al., 2017; Weissensteiner et al., 2017). Recombination is usually suppressed 5 to more than 200‐fold at centromeres (Rahn & Solari, 1986; reviewed in Talbert & Henikoff, 2010), such that linked selection has a more pronounced effect on nucleotide diversity (Burri, 2017; Cruickshank & Hahn, 2014). Both purifying selection against deleterious alleles (background selection; Charlesworth et al., 1993) and positive selection contribute to linked selection (Cutter & Payseur, 2013). In general, purifying selection is suggested to be more pervasive than positive selection (Burri, 2017), but several flycatcher (*Ficedula spp*.; Burri et al., 2015) and stonechat species (*Saxicola spp*.; Van Doren et al., 2017) show an excess of high‐frequency derived alleles (low values of Fay & Wu's H) at some of their putative centromeric regions, which indicates that they may be under positive selection (Fay & Wu, 2000). Albeit positive selection on centromeric regions could have many reasons, the signature of positive selection may also be caused by meiotic drive (Cruickshank & Hahn, 2014; Ellegren et al., 2012). Moreover, a second region of very low genetic diversity is found on most Australian zebra finch chromosomes, typically at the “distal end” that is about 50 cM away from the centromere (Knief et al., 2016) and these places might potentially evolve neocentromeric function (see above; Meyer et al., 2012).

Here, we investigated the idea that drive systems may evolve but become cryptic due to driver allele fixation or due to suppression of drive. We did this in two steps. First, we crossed two subspecies of zebra finches that have evolved in isolation for about two million years (Balakrishnan & Edwards, 2009): the Australian zebra finch (*T. g. castanotis*) and Timor zebra finch (*T. g. guttata*; Figure S1). The former has a remarkably large effective population size, while the latter is genetically much less diverse (Balakrishnan & Edwards, 2009). Thus, we hypothesized that, in the larger Australian population, selfish de novo mutations will have arisen and will have outcompeted the ancestral nondriving allele more often than in the Timor subspecies because there are more individuals in which such a mutation can arise.

Second, we produced a backcross to the Australian subspecies to monitor the performance of Timor centromeres in a predominantly Australian genetic background. Successful drive might be conditional on the protein machinery that controls the segregation of chromosomes. If the genetic background is essential for whether drive happens, we would expect to see differences between females in the genetically heterogeneous backcross generation (females carry 25% of Timor DNA on average, but this varies among individuals and between chromosomes). Hence, we also tested whether female identity significantly affects segregation ratios. To assay segregation distortion in hybrid females, we traced the genetic ancestry of 56 informative molecular markers in close linkage to centromeres and regions with neocentromeric potential (“distal ends”) in the zebra finch genome (Knief & Forstmeier, 2016) through a four‐generations backcross pedigree. As a control, we also estimated transmission ratios in hybrid males at centromeres and distal ends.

## 2 MATERIAL AND METHODS

### 2.1 Study populations

In 2013, we obtained four (2 males, 2 females) Timor zebra finches from a local breeder in Germany, two of which were brother and sister. The Australian zebra finches used in this study stemmed from a recently wild‐derived population housed at the Max Planck Institute for Ornithology in Seewiesen, Germany. They are descendants of birds from study population “Bielefeld‐AUS” described in Forstmeier et al. (2007) and genetically close to wild Australian birds.

The two subspecies differ phenotypically (Clayton et al., 1991, Figure 1). Timor zebra finches weigh less and have shorter wings than Australian zebra finches. Males also differ in that Timors have more orange (less red) beaks, smaller (absolute and relative to their size) black breast bands, and no black barring on the throat and foreneck. All these differences were found between the four Timor and 110 Australian zebra finches used in this study (all birds measured by the same observer, WF, Table S1).

**FIGURE 1:**
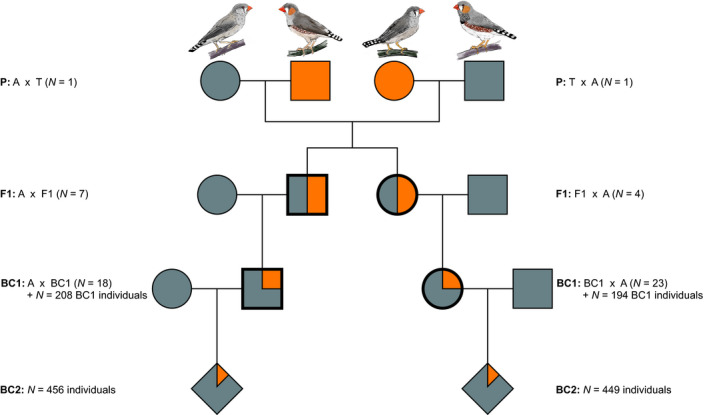
The breeding design and a description of the hybrid generations employed in this study. Squares represent males, circles females and diamonds a mixture of both sexes. Meioses in the four main groups (bold symbols) were informative for the analyses of meiotic drive. Colors refer to the expected fraction of Australian (gray) and Timor (orange) ancestry in each generation, respectively. Sample sizes refer to the numbers of breeding pairs and the combined numbers of embryos and offspring from the F1‐, BC1‐, and BC2‐hybrid generations. A, Australian zebra finch; T, Timor zebra finch

To test whether any admixture between Australian and Timor zebra finches had occurred in captivity prior to our experiment, we examined the genome of the male Timor zebra finch that bred in this study. This individual had been sequenced for another project (data kindly shared by Alexander Suh) with 60× coverage using Illumina paired‐end reads (150 bp; details of library preparation, analyses scripts, and data will be deposited along with that project). In brief, reads were quality‐ and adaptor‐trimmed using BBDuk (v38.25; https://sourceforge.net/projects/bbmap/), and then mapped against the reference genome WUSTL 3.2.4 (Warren et al., 2010) using bwa‐mem (v0.7.17; Li & Durbin, 2009). We called SNPs on this Timor male and also on 100 wild‐caught Australian zebra finches that had been sequenced using a pooled‐sequencing approach (see Knief, Hemmrich‐Stanisak, et al., 2015; Knief et al., 2016 for methodological details and the Open Science Framework [https://osf.io/dkqth/] for data) simultaneously with samtools (v1.6; Li et al., 2009) using “mpileup” (using a base quality Pfed score and a mapping quality score of more than 20 while removing reads that were unmapped, secondarily mapped, quality filtered or duplicated). This generated a set of 24 million high‐quality SNPs, in which we compared the SNPs of the Timor male with those in the pool of 100 wild‐caught Australian zebra finches. Specifically, we counted the number of SNPs that were homozygous (nonreference) in the Timor zebra finch and absent from the pooled‐sequencing data in 500 kb nonoverlapping sliding windows. We regarded those genomic windows that had less than 100 Timor‐specific SNPs as being introgressed from an Australian zebra finch. We identified 16 such regions (covering the centromeres on chromosomes *Tgu1*, *Tgu5*, *Tgu13* and *Tgu15*, Figure S2), all of which were heterozygous (i.e., 4.78% of the diploid genome is admixed). Hence, none of the 16 introgressed Australian regions were homozygous for the Australian haplotype, meaning that no potential meiotic drivers had gone to fixation prior to our study.

### 2.2 Breeding design

Birds were housed and bred in large semi‐outdoor aviaries. For a detailed description of the housing conditions, see Ihle et al. (2013). For breeding, individuals were allowed to freely choose partners. Individuals from each population were split according to sex and put in aviaries such that individuals with Timor ancestry could only pair with individuals from Australia (and vice versa; 2–4 aviaries with 4–24 birds each).

First, we hybridized Australian and Timor zebra finches to produce an F1 generation (Figure 1; *N* = 11 hybrid individuals that reached sexual maturity, 7 males and 4 females). Only two of the four Timor birds (one of each sex, not siblings) contributed to F1. Then, the F1 generation was backcrossed to Australian birds (BC1, Figure 1). Each pair was allowed to raise young from two clutches (*N* = 51 individuals that reached sexual maturity, 26 males and 25 females). Of those birds, 41 (18 males and 23 females) were again backcrossed to Australian birds (BC2, Figure 1).

From the BC1 generation, we sampled DNA from almost all eggs from all clutches, including dead embryos and deceased chicks. A small number of eggs were either broken (*N* = 3), lost (*N* = 3) or had no egg yolk (*N* = 1) but since this was a random subset of all eggs, it should not affect any of our conclusions. Eggs from clutches that were not used for further breeding were collected, put into an incubator and opened prior to hatching in order to obtain DNA samples. Thus, the number of hatched eggs reported below does not correspond to the actual hatching success. In total, we obtained 443 offspring samples (including the 51 surviving individuals). From the BC2 generation, most of the eggs were sampled for DNA but a small number of eggs were again broken (*N* = 8) or lost (*N* = 22). This yielded a total of 905 offspring samples (*N* = 79 individuals that reached sexual maturity and *N* = 826 embryos or dead chicks). We doubled the sample size compared to the previous generation to reach similar power in tests for meiotic drive across generations. This is necessary because all birds from the F1 generation are heterozygous with respect to the Australian and Timor ancestry for their entire genome, while in the BC1 generation, only half of the chromosomal combinations are expected to be admixed.

Problematically, captive zebra finches lay a considerable proportion of eggs (typically 10%–40% [Pei et al., 2020], here 12%) that appear to be infertile (no visible embryo development). In wild Australian zebra finches, around 17% of all eggs fail to hatch (Griffith et al., 2008; Mariette & Griffith, 2012; Zann, 1996). Studies that have examined the presence of sperm on the perivitelline layer of eggs (Birkhead & Fletcher, 1998; Pei et al., 2020) revealed that it is nearly always the absence of sperm that explains why an egg is not fertilized. However, the risk remains that at least some of the eggs classified as infertile suffered early embryo mortality, such that the development cannot be seen, a problem that can be dealt with in various ways (see Knief, Schielzeth, et al., 2015). To control for such potential early embryo mortality, we here contrasted the transmission ratios in female parents to those in male parents, because we did not expect any drive linked to centromeres or distal ends in the latter (see below).

Our backcross design was asymmetric (F1 hybrids were backcrossed to Australian birds but not to Timor birds) for the following reasons. First, only for Australian birds did we have a large enough and genetically diverse captive population available for backcrossing. This is needed to avoid high rates of early embryo mortality due to inbreeding (Bolund et al., 2010; Forstmeier et al., 2012). Second, it is more likely that a driving allele has gone to fixation in the subspecies with the larger effective population size in the wild (i.e., the Australian, see Introduction). Such drivers might require the segregation “machinery” of the Australian subspecies to be active. Hence, backcrossing hybrids to Australian birds will more likely expose cryptic drivers, if they exist.

Because uncontrolled introgression of Timor DNA into captive Australian zebra finch populations is undesirable, all hybrids were sacrificed at the end of this study. All procedures on zebra finches (housing, breeding, banding, bleeding for parentage assignment, measuring, observing, and sacrificing) do not qualify as animal experimentation according to the relevant national and regional laws and are fully covered by our housing and breeding permit (# 311.4‐si, by Landratsamt Starnberg, Germany).

### 2.3 Genetic markers

Previously, we used a set of 62 microsatellite markers to infer the positions of centromeres in the current zebra finch genome assembly (WUSTL 3.2.4; Warren et al., 2010) via half‐tetrad mapping (Knief & Forstmeier, 2016). On each assembled chromosome, there was one marker linked to the centromere and one at the distal chromosomal end. For this study, we genotyped all individuals for all 62 microsatellite markers. However, we removed six markers because (1) they were duplicated in the genome (marker 12_en_20.79), (2) genotyping failed (markers 1B_st_0.19 and 17_en_11.11), or (3) the centromere had not been positioned unambiguously (markers 1B_en_1.05, 27_st_0.58 and 27_en_4.57 on chromosomes *Tgu1B* and *Tgu27*). A detailed description of the DNA extraction, the 62 microsatellite markers, and genotyping procedures is given in Knief and Forstmeier (2016).

Thus, we genotyped all samples for 29 markers linked to the centromere and 27 markers at the distal end of all assembled chromosomes except *Tgu1B*, *Tgu27* (and 13 unassembled microchromosomes). The microsatellites linked to the centromere were maximally 27 cM (median 0 cM) and the unlinked markers at least 18 cM (median 50 cM) away from the centromere as determined via half‐tetrad mapping (Knief & Forstmeier, 2016). The minimum distance between markers was 17 cM. Genetic distances of less than 50 cM between centromeres and unlinked markers are mostly found on the seven (sub)metacentric chromosomes (*Tgu1*, *Tgu1A*, *Tgu2*, *Tgu3*, *Tgu4*, *Tgu7,* and *TguZ*), where there is not always one crossover per chromosome arm per meiosis (Calderón & Pigozzi, 2006).

### 2.4 Analysis of parentage & chromosomal abnormalities

We performed genetic parentage analyses for each aviary separately using the SOLOMON package (v1.0‐1; Christie et al., 2013) in R (v3.4.3; R Core Team, 2017). We used the “Bayesian parentage analysis with no known parents” option with the recommended default settings for microsatellite genotype data. Using all 56 microsatellite markers, parents were unambiguously assigned to all offspring.

For embryos with chromosomal abnormalities (*N* = 39 out of a total of 1,359 samples; showing either tetraploidy, triploidy, haploidy, trisomy, or monosomy), we identified the parental origin and the most likely cause of the error (nondisjunction in meiosis I or in meiosis II or polyspermy). Whenever the parent contributing the abnormal chromosome set was a hybrid or a backcrossed individual (whose inheritance of alleles is of interest here), we removed the offspring with the chromosomal abnormality from further analyses for the chromosomes affected.

### 2.5 Testing for segregation distortion

We followed the Timor and the Australian microsatellite alleles through the backcross pedigree and measured segregation distortion among alleles transmitted by birds of the F1 and BC1 generations (Figure 1). Whenever an individual was heterozygous for an Australian and a Timor allele with different microsatellite lengths, we counted how many times the individual passed on the Australian and the Timor allele to its offspring. Note that our classification of alleles as being of Australian or Timor origin assumed no introgression prior to our experiment. After the completion of our study, we discovered a few introgressed Australian alleles (about 5% of genome, see above) in one of the Timor founders. This introduces some error into our classification, which could only be eliminated if all founders would have been sequenced. Given that our study revealed no clear positive support for the existence of a specific driver, we decided against eliminating this source of error.

Heterozygous genotypes with null alleles (i.e., allelic drop‐outs that were inferred from the pedigree) were excluded. Because we observed no sex‐ratio bias (female–male ratio, FMR) in the backcross generations (BC1: FMR (±*SE*) = 1.09 (0.99–1.20), *p* = .37; BC2: FMR = 1.06 (1.00–1.14), *p* = .35; BC1 + BC2: FMR = 1.07 (1.02–1.13), *p* = .20), we coded the female‐specific *TguW* chromosome as a null‐allele, such that we effectively tested for transmission distortion in the homogametic males only. See Table S2 for a detailed description of all possible genotype combinations and how they were treated in the analyses. Importantly, whenever for a particular locus a set of parents carried alleles that were fully informative about their origin (stemming from Australia vs. Timor) irrespective of offspring genotype, all their offspring were included in the analysis. In contrast, when the origin could only be identified for certain allelic constellations in the offspring, all offspring from such pairs were excluded, except for the parental constellation AB × AB, where we included homozygous offspring (*N* = 330 informative transmissions of which 164 were an Australian and 166 a Timor allele; see Table S2 for more details). We counted the transmissions for all markers (centromeres and distal chromosome end), for both females and males, and for the F1 and BC1 generation separately. We expected a chromosomal meiotic driver to act only in female parents at centromeres (drive in meiosis I) or at distal ends (drive in meiosis II).

Throughout the study, we counted the transmission of Australian alleles as 1 (success) and of Timor alleles as 0 (failure). We define the drive parameter *k* as the proportion of progeny (successful gametes) that inherited an Australian allele (see also Lyttle, 1991). Thus, at fair Mendelian segregation *k* = 0.5 and when Australian alleles are more often transmitted than Timor alleles *k* > 0.5.

To obtain estimates of the background transmission rates, we fitted generalized linear mixed‐effects models (GLMM) with a binomial error structure using the transmission counts of all markers (1 = Australian allele, 0 = Timor allele transmitted) as the dependent variable, the intercept as the only fixed effect, and marker identity as a random intercept effect. We fitted separate models for females and males and for both sexes combined. We used the *k*‐value estimate across all markers in male parents as the background transmission rate, which makes all estimates in our study more conservative because *k* was biased in the same direction across generations and sexes. Finally, we fitted a GLMM using data from all generations, markers and both sexes, in which we fitted a dummy variable (generation [F1, BC1] × chromosomal position [centromeric, distal] × sex [female, male]; eight levels) as a fixed effect and marker identity as a random intercept effect.

We then tested the empirical transmission ratios of individual markers in female parents against the background transmission rate (null hypothesis) by fitting generalized linear models (GLM) with a binomial error structure using the transmission count (1 = Australian allele, 0 = Timor allele transmitted) as the dependent variable and the intercept as the sole predictor. Here and in all following models, we specified the background transmission rate as an offset term. This is equivalent to using a binomial test (as in Knief, Schielzeth, et al., 2015) with a success probability of the background transmission rate. We report drive parameter *k* estimates from these models, such that *k* = 0.5 corresponds to the background transmission rate (background *k* = 0.495, see results) and *k* < 0.5 describe drive parameters smaller than the background rate. By considering all markers separately, weak genome‐wide drive could be missed and we thus fitted the same GLMMs as described above for female parents and with the eight categories, but this time controlling for the background transmission rate. If meiotic drivers are only functional when parts of their proteinaceous meiotic machinery have an entire Australian ancestry (i.e., they are homozygous for an Australian genetic background for some crucial loci), then this could be observed in birds from the BC1 generation, which are partially heterozygous and partially homozygous with respect to their genetic ancestry. We thus also fitted GLMMs with a binomial error structure using the transmission counts of all markers (1 = Australian allele, 0 = Timor allele transmitted) in birds from the BC1 generation as the dependent variable, the intercept as the only fixed effect, and individual identity as a random intercept effect, while controlling for the background transmission rate. If the random effect differed from 0 then there would be more variation among individuals than expected by chance and thus potentially a meiotic driver that was only active in some individuals with the drive‐enabling genetic background.

GLMs were fitted in R (v3.4.3) using the glm() function and GLMMs using the glmmadmb() function from the glmmADMB package (v0.8.3.3; Fournier et al., 2012; Skaug et al., 2016), which allows testing the significance of single random effects via likelihood ratio tests (LRT). *p*‐Values were not corrected for multiple testing. We back‐transformed all GLM and GLMM estimates using the invlogit() function of the arm package (v1.9‐3; Gelman & Su, 2016) in R. Power analyses were performed using the pwr.p.test() function from the pwr package (v1.2‐1; Champely, 2017) in R (v3.4.3). For convenience, we report *k* > 0.5 in the power analyses, but it should be noted that *k* is symmetric around 0.5. All data can be found in Knief et al. (2019).

## 3 RESULTS

We determined 74,829 microsatellite allele transmissions in the F1 and BC1 generations, of which 38,541 (51.5%) were informative for estimating transmission ratios of Australian and Timor alleles. Sample sizes were evenly distributed across sexes and generations (Table S3), but varied between markers depending on their allelic diversity, heterozygosity, and null‐allele frequency within the population (all *p* ≤ .01; Table S4). When pooling all markers within generations and sexes, power to detect even weak deviations from Mendelian segregation was high (range of lower bound *k* = 0.517–0.519 [or *k* = 0.481–0.483] with 80% power). For individual microsatellites in female parents, we could identify deviations from Mendelian segregation ratios ranging from a lower bound *k* = 0.553 [0.447] to *k* = 0.642 [0.358] (mean lower bound *k* = 0.568 [0.432]) with 80% power at a nominal *p*‐value of .05.

**FIGURE 2:**
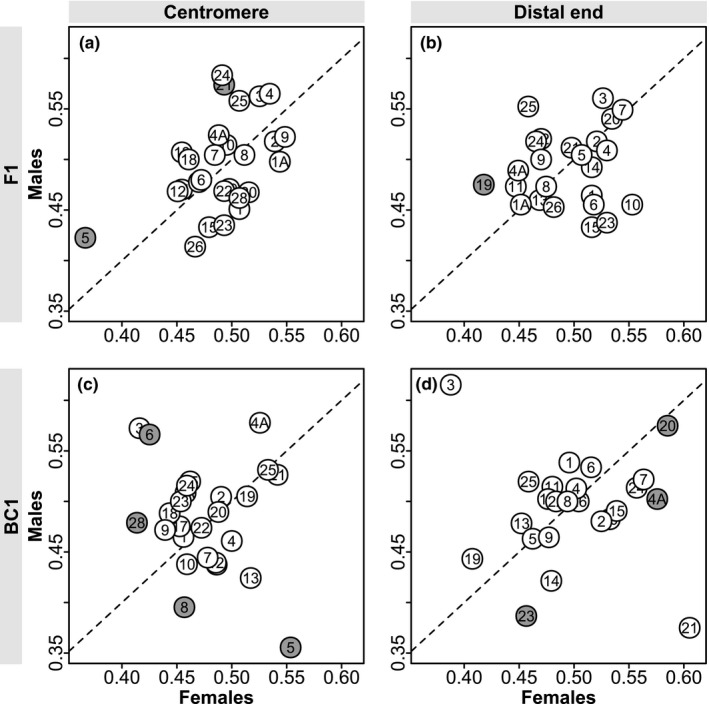
Transmission ratios of individual markers in the F1 generation (a,b) and in the BC1 generation (c,d) at centromeres (a,c) and distal chromosomal ends (b,d). Transmission ratios in females and males are plotted on the x‐ and y‐axis, respectively. Numbers refer to the chromosome number in the zebra finch genome assembly and a gray background indicates a significant deviation from fair Mendelian segregation in either of the two sexes (no marker deviated significantly in both sexes). Note that only 11 out of 208 tests reached statistical significance at α = 0.05. The dashed line is the identity line

**FIGURE 3:**
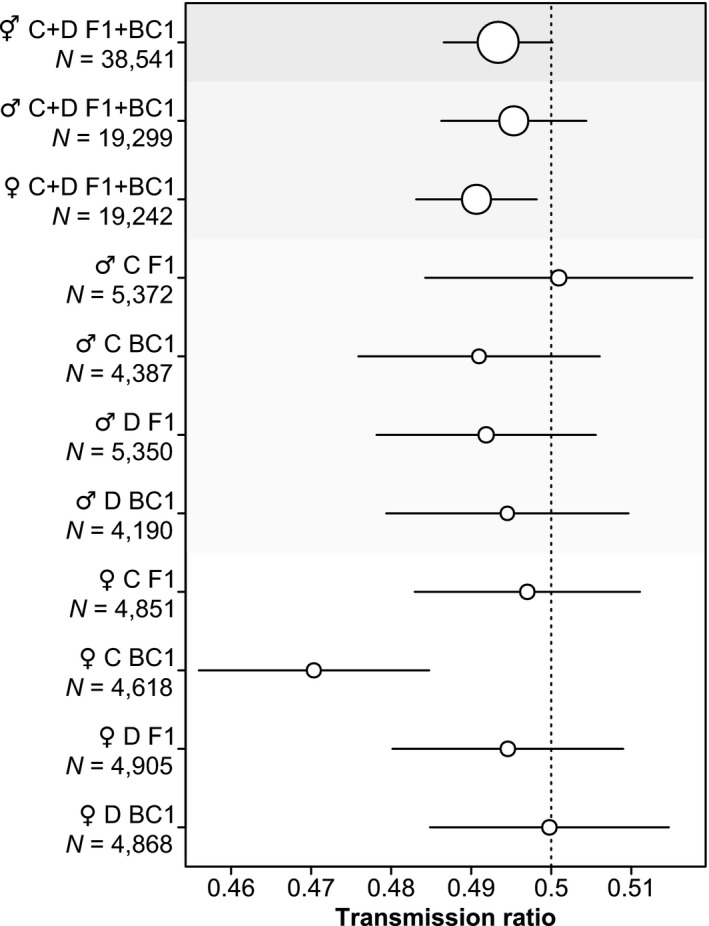
Summary of transmission ratios ±95% confidence intervals for all markers. C, centromeric markers; D, distal markers, F1 and BC1 refer to the generation (see Figure 1). Point size reflects sample size, that is, the number of informative meioses (*N*). All estimates stem from models in which the background transmission rate was not accounted for

Across all markers, generations and sexes there was a small bias toward an increased transmission of Timor alleles (GLMM: *N* = 38,541 informative transmissions, *k* = 0.493, *p* = .056 [GLM: *p* = .0044]; Figures 2 and 3, Table S5: “All markers”), which was similar for mothers and fathers, although only significant for the former (GLMM females: *N* = 19,242 informative transmissions, *k* = 0.491, *p* = .015 [GLM: *p* = .0095]; GLMM males: *N* = 19,299 informative transmissions, *k* = 0.495, *p* = .31 [GLM: *p* = .15]; Figure 3, Table S5: “All markers”). We ruled out that this bias resulted from undetected null alleles by analyzing the subset of trios where both parents were heterozygous without a null‐allele (combinations 4, 7, and 13 in Table S2). The effect did not change (combined sexes GLMM: *N* = 25,685 informative transmissions, *k* = 0.491, *p* = .014; Table S5: “Heterozygous parents”). Next, we tested whether inbreeding depression might have caused this deviation from Mendelian segregation by sub‐setting the data to those trios in which the parents could not produce offspring homozygous for an Australian microsatellite allele (combinations 11, 12, 13, and 16 in Table S2). We thereby assumed that microsatellites tagged larger haplotypes in our captive population that became identical‐by‐descent in individuals homozygous for a specific Australian allele. In this dataset, the bias toward an increased transmission of Timor alleles was slightly lower and nonsignificant (GLMM: *N* = 18,714 informative transmissions, *k* = 0.496, *p* = .27; Table S5: “No inbreeding”). Thus, to rule out inbreeding effects, we set the transmission rate estimated from all markers in male parents (*k* = 0.495) as the background transmission rate against which we tested transmission in females.

None of the markers linked to centromeres or distal chromosomal ends showed significant deviations from fair Mendelian segregation in females, if we would have applied Bonferroni correction (GLM: *N* = 27 markers with informative transmissions linked to centromeres, *k* = 0.452–0.524, all nominal *p* ≥ .038; GLM: *N* = 24 markers with informative transmissions linked to telomeres, *k* = 0.421–0.559, all nominal *p* ≥ .010; Table S6). Analyzing all centromeric markers in female parents combined, there was a slight bias toward an increased transmission of Timor alleles (GLMM: *N* = 9,469 informative transmissions, *k* = 0.489 [which is a deviation of 0.011 from the background rate of 0.495], *p* = .035 [GLM: *p* = .035]; Figure 3), whereas the markers at the distal chromosomal end showed no deviation from Mendelian segregation (GLMM: *N* = 9,773 informative transmission, *k* = 0.502, *p* = .71 [GLM: *p* = .66]; Figure 3, Table S5: “All markers”). The bias in centromeric marker transmissions was only present in the BC1 generation (*k* = 0.475, *p* = 9 × 10^–4^, Figure 3, Table S5: “All markers”), which was significantly different from all other combinations of generation [F1, BC1] × chromosomal position [centromeric, distal] × sex [female, male] (*p* = .014; Figure 3, Table S5: “All markers”). However, we found no evidence for our hypothesis that a cryptic driver would be unleashed in only some individuals with a predominantly homozygous Australian genetic background, because in the BC1 generation there was no significant effect of female identity (random effect) on transmission ratios at centromeric (GLMM: LRT *p* = .81; Table S7) or distal chromosomal markers (GLMM: LRT *p* = .58; Table S7). It seems unlikely that the apparent overall drive at centromeric markers in BC1 females was due to undetected early embryo mortality (i.e., the missing Australian alleles being hidden in eggs that were incorrectly judged as infertile), because the overall rate of apparent infertility was low (only 6% of eggs) when BC1 females were crossed with Australian males in comparison to other crosses (Table S8).

## 4 DISCUSSION

Most of the meiotic drivers discovered thus far exhibit drive parameters of *k* > 0.60, especially in crosses between populations or species (Chmátal et al., 2014; Didion et al., 2015; Fishman & Saunders, 2008; Fishman & Willis, 2005; Rhoades, 1942). Despite having high power to discover even smaller deviations from Mendelian segregation in our study, we found no clear evidence for any active meiotic driver in a cross between Australian and Timor zebra finches. It should be noted, however, that we tagged centromeres of only 27 chromosomes, whereas the somatic zebra finch genome consists of 40 chromosomes (Pigozzi & Solari, 1998), leaving the possibility for drivers on the remaining—as yet mostly unassembled—13 microchromosomes.

We found a weak deviation from fair Mendelian segregation ratios in both female and male parents at both centromeres and distal chromosomal ends, indicating the presence of a selective force other than meiotic drive. Contrary to our expectation that the more efficient drive alleles would have evolved in Australian zebra finches, the bias was in favor of Timor alleles being more often transmitted to the next generation. We used a breeding design in which we backcrossed hybrids to Australian zebra finches. Because of the low recombination rate in the zebra finch genome (Backström et al., 2010), this could have led to large chromosomal parts becoming identical‐by‐descent. The resulting inbreeding depression might have manifested itself through increased early embryo mortality without any visible embryonic development (Bolund et al., 2010; Forstmeier et al., 2012). If so, it would appear as if Australian alleles were transmitted less often than Timor alleles to the surviving offspring. In contrast, Bateson‐Dobzhansky‐Muller (BDM; Bateson, 1909; Dobzhansky, 1937; Muller, 1942) incompatibilities are more likely to remove admixed individuals and thus would have caused deviations toward Australian alleles in the offspring. The manifestation of Haldane's rule—itself explained as a form of BDM (Schilthuizen et al., 2011)—relevant for this study would have been a biased sex‐ratio, which we did not observe (female‐male ratio [FMR] across both backcross generations: FMR [±*SE*] = 1.07 [1.02–1.13]). Furthermore, because BDMs depend on specific interactions between loci, only some parents would inherit the detrimental allelic combinations. Thus, BDMs are further expected to cause variation in transmission ratios between parental individuals, which we did not find. Specifically testing inbreeding and BDM incompatibility effects ideally requires a reciprocal backcross design.

After accounting for the above‐mentioned small deviation from fair Mendelian segregation that might have been caused by inbreeding, none of the chromosomes exhibited segregation distortion by themselves. However, we still observed a small deviation from Mendelian segregation in females of the BC1 generation at centromeres, again in the direction of an increased transmission of Timor alleles (*k* = 0.470 without and *k* = 0.475 with control for the background transmission rate). Tests for meiotic drive are sensitive to genotyping errors (see Meyer et al., 2012), which we ruled out by using the transmission ratios of the same markers in males as our background transmission rate. Given that there was also no such effect at distal chromosomal ends in females, this might indicate weak genome‐wide meiotic drive in meiosis I, in which Timor centromeres preferentially enter the oocyte and outcompete the Australian alleles. This could happen if larger centromeres are preferentially transmitted within a specific genetic background. Many small mutations may accumulate in such a species, leading to all or most centromeres being enlarged. Whenever these enlarged centromeres compete with shorter centromeres (e.g., in a female hybrid), this would result in genome‐wide meiotic drive, as has been found in mice (Akera et al., 2019; Iwata‐Otsubo et al., 2017). The nobs in maize are another example, which can be present on all chromosomes, show neocentromeric activity, and—in the heterozygous state—drive across many chromosomes (Rhoades, 1942).

In pure Australian zebra finches, we had previously observed a potential meiotic driver on chromosome *Tgu5* (*k* = 0.602) that was active in only some generations of an extended pedigree (Knief, Schielzeth, et al., 2015). Together with the current results, this might indicate that some meiotic drivers are environmentally induced and not constantly active, as has been described in other systems (Rhoades, 1942; Shaw & Hewitt, 1984). Alternatively, such cases may be examples of the “winner's curse” (Xiao & Boehnke, 2009), in which a false‐positive finding is followed by true‐negative results.

## 5 CONCLUSION

Meiotic drive has been proposed as a potent evolutionary force but its frequency in nature remains unknown and its impact on genetic and phenotypic variation, genome structure, or speciation is difficult to assess. We here specifically tested for deviations from fair Mendelian segregation of chromosomes in a cross between two diverged subspecies, the Australian and Timor zebra finch. Crossing phylogenetically more distant species to Australian zebra finches does not result in fertile offspring (Forshaw et al., 2012; McCarthy, 2006). We expected the more potent meiotic drivers to evolve in the population with the larger effective population size, which is the Australian zebra finch. However, although the weak genome‐wide segregation distortion that we observed in females of the BC1 generation might indeed be attributable to centromeric meiotic drive, the Timor alleles outcompeted the Australian ones. Thus, we caution against interpreting our finding as definite evidence for centromeric drive. A nominal P‐value of 6 × 10^–5^ is unlikely a type I error, but not fully compelling either, because one could argue that we tested 56 markers in two generations, two sexes and with and without taking the background transmission rate into account (56 × 2 × 2 × 2 = 448 tests, translating into a Bonferroni corrected *p*‐value ≈ .03).

We failed to find evidence for strong localized drivers. The moderate driver on chromosome *Tgu2* that we had observed previously (Knief, Schielzeth, et al., 2015) in a domesticated population of Australian zebra finches (population “Seewiesen‐GB” in Forstmeier et al., 2007) was not present in the pedigree we analyzed for the current study (Australian zebra finches were derived from population “Bielefeld‐AUS” in Forstmeier et al., 2007).

Assuming an infinite population size and no heterozygote disadvantage, even weak drivers will eventually invade and ultimately reach fixation (Traulsen & Reed, 2012), thereby causing a reduction in genetic diversity at the driving loci and potentially a decrease in organismal fitness (“selfish sweeps”; Didion et al., 2016). Weak meiotic drivers as we might have found here have not been reported yet, but this might in part be due to detection bias and insufficient statistical power. Weak meiotic drive might be a more common phenomenon warranting further investigation in other taxa.

## CONFLICT OF INTEREST

The authors declare that they have no competing interests.

## AUTHOR CONTRIBUTION

**Ulrich Knief:** Conceptualization (equal); Data curation (lead); Formal analysis (lead); Methodology (equal); Software (lead); Validation (lead); Visualization (lead); Writing‐original draft (lead); Writing‐review & editing (equal). **Wolfgang Forstmeier:** Conceptualization (lead); Formal analysis (supporting); Investigation (equal); Methodology (equal); Project administration (lead); Supervision (lead); Writing‐original draft (equal); Writing‐review & editing (lead). **Yifan Pei:** Formal analysis (supporting); Visualization (supporting). **Jochen Wolf:** Conceptualization (supporting); Resources (supporting); Supervision (supporting); Writing‐review & editing (equal). **Bart Kempenaers:** Conceptualization (supporting); Funding acquisition (lead); Resources (lead); Writing‐original draft (equal); Writing‐review & editing (equal).

### Open Research Badges

This article has been awarded Open Materials, Open Data Badges. All materials and data are publicly accessible via the Open Science Framework at https://doi.org/10.17605/osf.io/52evp and Open Science Framework (https://osf.io/dkqth/).

## Supporting information

Supplementary Material

## Data Availability

Data on all individuals used in the current study, their genotypes and the R‐script used for inferring ancestry of all genotypes are accessible through the Open Science Framework (https://doi.org/10.17605/osf.io/52evp).
