# Supplementary material for: A test for meiotic drive in hybrids between Australian and Timor zebra finches
Source: Ecol Evol. 2020 Nov 3;10(23):13464–75. doi: 10.1002/ece3.6951 (PMC7713956; doi:10.1002/ece3.6951)
Supplement: Supplementary file 1 — Supplementary Material [file ECE3-10-13464-s001.pdf]

SUPPLEMENTARY MATERIAL

A test for meiotic drive in hybrids between Australian and Timor zebra finches

Ulrich Knief[1,2,*], Wolfgang Forstmeier[1], Yifan Pei[1], Jochen Wolf[2], Bart Kempenaers[1]

[1] Department of Behavioural Ecology and Evolutionary Genetics, Max Planck Institute for Ornithology, 82319 Seewiesen, Germany

[2] Division of Evolutionary Biology, Faculty of Biology, Ludwig Maximilian University of Munich, 82152 Planegg-Martinsried, Germany

[*] Address for correspondence: Ulrich Knief, Division of Evolutionary Biology, Faculty of Biology, Ludwig Maximilian University of Munich, Grosshaderner Str. 2, 82152 Planegg-Martinsried, Germany, Phone: 0049-89-2180-74101, Fax: 0049-89-2180-74104, E-mail: knief@biologie.uni-muenchen.de

Index

Supplementary Figures **2**
Supplementary Tables **4**
References **26**

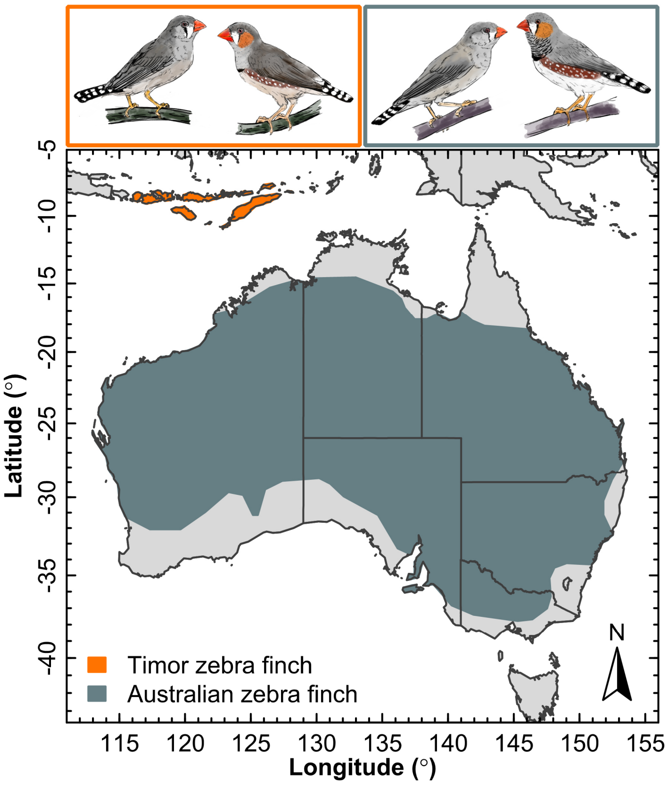

Figure S1 | Illustration and distribution map of the Timor and the Australian zebra finch (*Taeniopygia guttata guttata* and *T. g. castanotis*, respectively). Data were kindly provided by BirdLife International and Handbook of the Birds of the World (2016a, b).

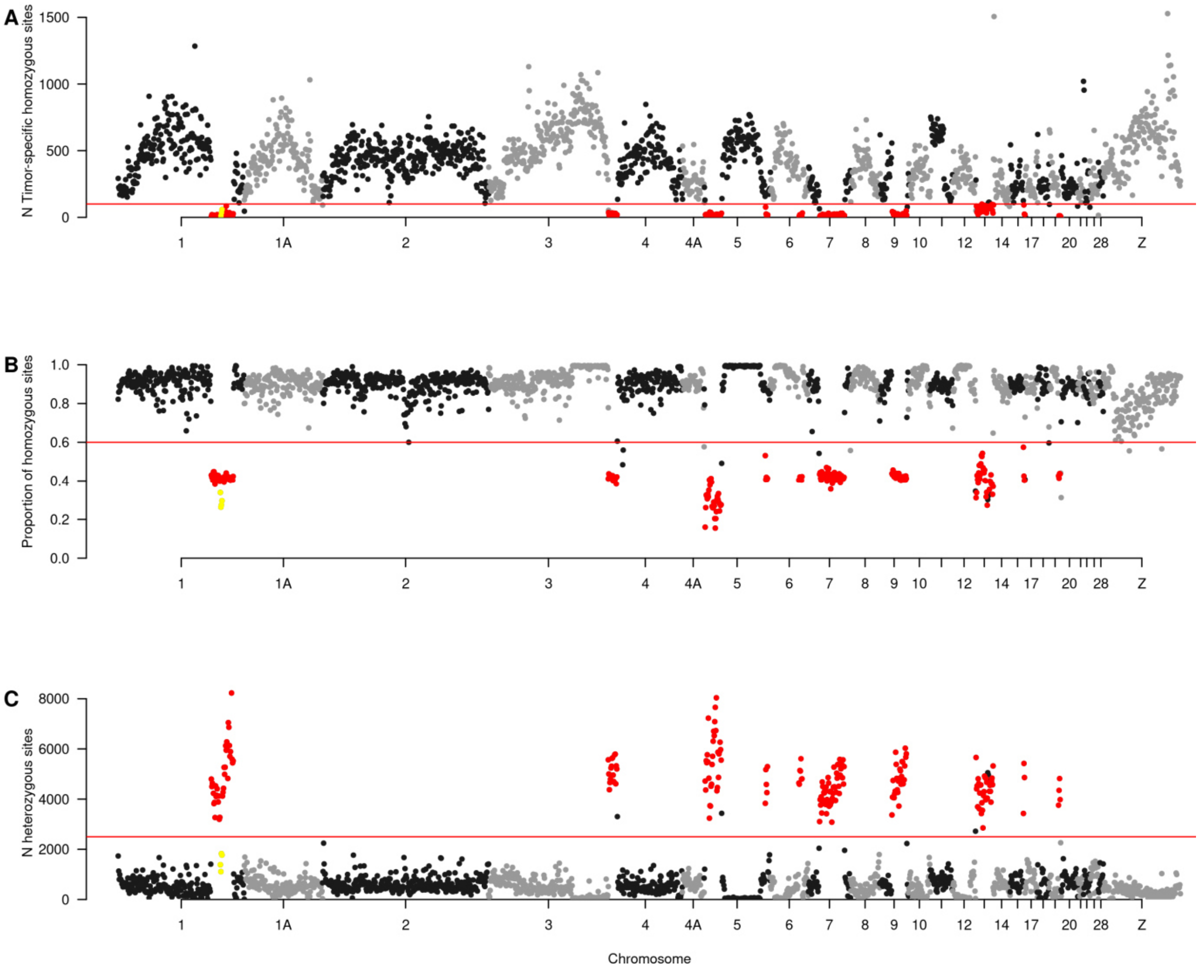

Figure S2 | Admixture between Timor and Australian zebra finches prior to the current experiment as estimated from genomic data from the male Timor zebra finch breeding in this study. (A) Number of homozygous sites that are fixed in this Timor zebra finch and absent from the 100 wild-caught Australian zebra finches, (B) proportion of homozygous sites and (C) number of heterozygous sites in 500 kb non-overlapping sliding windows. Red highlights windows that have less than 100 fixed Timor-specific homozygous sites in (A), a proportion of less than 0.6 homozygous sites in (B) and more than 2500 heterozygous sites for a specific window in (C), as indicated by the red horizontal cut-off lines. Yellow on chromosome *Tgu1* highlights windows that stood out in (A) and (B) but not in (C). Note that this yellow region contains the centromere of chromosome *Tgu1*.

Table S1 | Phenotypic differences between Australian and Timor zebra finches. Mixed-effects models were fitted using the pedigreemm R-package (v0.3-3; Vazquez *et al.* 2010), which takes relatedness between individuals into account.

Phenotype	Parameter	Estimate	SE	z-value	P-value
Body mass (g)	Australia	11.55	0.14		
	Timor	-1.95	0.47	-4.18	6×10^{-5}
Wing length (mm)	Australia	55.29	0.25		
	Timor	-3.75	0.75	-4.98	3×10^{-6}
Beak colour (1-5)	Australia	3.95	0.05		
	Timor	-1.30	0.17	-7.50	9×10^{-10}
Breast band size (0-5)	Australia	2.11	0.1		
	Timor	-0.86	0.52	-1.64	0.098

Table S2 | All possible microsatellite genotype combinations with indication of whether they are useful (informative) for tracing genetic ancestry through the pedigree when breeding the F1 or assessing segregation distortion in the backcross generations. There can be at maximum four different microsatellite alleles within a parent-offspring trio (A, B, C and D or a null-allele 0). The focal individual is the offspring of either a Timor and an Australian zebra finch parent (breeding the F1) or a hybrid and an Australian parent (breeding the BC1 and BC2).

**Combination**	**Genotypes**			**Informative in breeding the F1**	**Informative for assessing segregation distortion in the backcrosses**
	**Focal individual**	**Timor/Hybrid parent**	**Australia parent**		
1	AA	AA	AA	0	0
2	A0	AA	A0	0	0
2	AA	AA	A0	0	0
2	A0	A0	AA	0	0
2	AA	A0	AA	0	0
3	AA	A0	A0	0	0
3	A0	A0	A0	0	0
3	00	A0	A0	1	0
4	AA	AB	AB	1	1
4	AB	AB	AB	0	0
4	BB	AB	AB	1	1
5	AA	AA	AB	1	0
5	AB	AA	AB	1	0
5	AA	AB	AA	1	1
5	AB	AB	AA	1	1
6	AA	A0	AB	0	0
6	AB	A0	AB	1	0
6	A0	A0	AB	0	0
6	B0	A0	AB	1	0
6	AA	AB	A0	1	1
6	AB	AB	A0	1	1
6	A0	AB	A0	1	1
6	B0	AB	A0	1	1
7	AA	AB	AC	1	1
7	AC	AB	AC	1	1
7	AB	AB	AC	1	1
7	BC	AB	AC	1	1
8	AB	AA	BB	1	0
9	AB	A0	BB	1	0
9	B0	A0	BB	1	0
9	AB	AA	B0	1	0
9	A0	AA	B0	1	0
10	AB	A0	B0	1	0
10	A0	A0	B0	1	0
10	B0	A0	B0	1	0
10	00	A0	B0	1	0
11	AB	AA	BC	1	0
11	AC	AA	BC	1	0
11	AC	AB	CC	1	1
11	BC	AB	CC	1	1
12	AB	A0	BC	1	0
12	AC	A0	BC	1	0
12	B0	A0	BC	1	0
12	C0	A0	BC	1	0
12	AC	AB	C0	1	1

12	BC	AB	C0	1	1
12	A0	AB	C0	1	1
12	B0	AB	C0	1	1
13	AC	AB	CD	1	1
13	AD	AB	CD	1	1
13	BC	AB	CD	1	1
13	BD	AB	CD	1	1
14	00	00	00	1	0
15	A0	AA	00	1	0
15	A0	00	AA	1	0
16	A0	AB	00	1	1
16	B0	AB	00	1	1
16	A0	00	AB	1	0
16	B0	00	AB	1	0
17	A0	A0	00	1	0
17	00	A0	00	1	0
17	A0	00	A0	1	0
17	00	00	A0	1	0

Table S3 | Total numbers of informative meioses per generation, sex and chromosomal region.

**Generation**	**Sex**	***N* Centromeres**	***N* Distal chromosome end**
F1	Female	4851	4905
F1	Male	5372	5350
BC1	Female	4618	4868
BC1	Male	4387	4190

Table S4 | Summary statistics for the 29 microsatellite markers linked to the centromere and 27 markers at the distal chromosomal end. Physical and genetic positions were taken from Knief & Forstmeier (2016). The physical position is in genome assembly WUSTL 3.2.4 (Warren *et al.* 2010) and the genetic position and its standard deviation is the genetic distance to the centromere. All other values were calculated from individuals in the BC1 and BC2 generation. H_O = observed heterozygosity; H_E = expected heterozygosity assuming random mating. Values for markers on the sex chromosome *TguZ* were calculated using males only. *N* alleles, Null-allele frequency and H_O were correlated with ln(*N* informative transmissions) ($r = 0.47$, $P = 3 \times 10^{-4}$, $r = -0.33$, $P = 0.013$, $r = 0.56$, $P = 7 \times 10^{-6}$, respectively; all $N = 55$, marker 14_st_1.07 was removed because it did not have any informative transmissions).

Microsatellite marker	Chromosomal side	Position (cM ± SD)	Position (Mb)	*N* alleles	Null-allele frequency	H_O	H_E	H_E-H_O	N informative transmissions
1_cen_98.17	Centromeric	0.00	98.17	8	0	0.756	0.709	-0.047	808
1A_cen_62.53	Centromeric	0.00	62.53	11	0	0.933	0.858	-0.074	945
2_cen_76.29	Centromeric	0.00	76.29	8	0	0.814	0.761	-0.054	978
3_cen_40.34	Centromeric	0.00	40.34	10	0	0.902	0.840	-0.062	711
4_cen_16.82	Centromeric	0.00	16.82	10	0.012	0.869	0.835	-0.034	765
4A_en_19.79	Centromeric	0.00	19.79	5	0	0.715	0.685	-0.030	566
5rand_cen_0.26	Centromeric	0.00	0.26	11	0.067	0.713	0.745	0.032	393
6_cen_0.89	Centromeric	0.00	0.89	9	0	0.920	0.859	-0.060	829
7_cen_4.65	Centromeric	0.00	4.65	13	0	0.924	0.885	-0.039	512
8_cen_1.38	Centromeric	4.65 ± 4.43	1.38	11	0.013	0.892	0.856	-0.036	767
9_st_0.96	Centromeric	0.00	0.96	4	0	0.408	0.363	-0.045	915
10_st_0.86	Centromeric	0.00	0.86	9	0.092	0.832	0.810	-0.022	816
11_en_20.8	Centromeric	0.00	20.8	7	0.021	0.766	0.706	-0.060	346
12_st_0.77	Centromeric	0.00	0.77	7	0	0.802	0.762	-0.041	603
13_en_16.75	Centromeric	27.04 ± 8.08	16.75	6	0.083	0.611	0.657	0.046	246
14_st_1.07	Centromeric	0.00	1.07	9	0.121	0.762	0.784	0.022	0
15_en_13.76	Centromeric	0.00	13.76	10	0	0.902	0.842	-0.060	1022
17_st_0.7	Centromeric	0.00	0.7	11	0	0.855	0.812	-0.043	854
18_st_0.48	Centromeric	0.00	0.48	9	0	0.899	0.869	-0.030	834
19_st_0.97	Centromeric	0.00	0.97	8	0	0.872	0.809	-0.062	891
20_st_1.91	Centromeric	0.00	1.91	6	0	0.841	0.782	-0.060	311
21_st_0.22	Centromeric	8.53 ± 5.63	0.22	9	0	0.924	0.853	-0.071	750
22_en_3.1	Centromeric	0.00	3.1	6	0.004	0.599	0.551	-0.048	834
23_st_0.68	Centromeric	0.00	0.68	14	0	0.884	0.871	-0.012	963
24_en_7.89	Centromeric	0.00	7.89	7	0	0.711	0.690	-0.021	441
25_st_0.03	Centromeric	5.31 ± 5.17	0.03	11	0	0.809	0.824	0.015	847
26_st_0.2	Centromeric	0.00	0.2	6	0	0.683	0.643	-0.040	104
28_st_0.29	Centromeric	0.00	0.29	8	0	0.903	0.839	-0.064	950
Z_cen_27.51	Centromeric	0.00	27.51	6	0.003	0.568	0.518	-0.050	227
1_st_0.48	Distal	18.28 ± 7.72	0.48	11	0.089	0.920	0.874	-0.046	753
1A_st_0.38	Distal	35.84 ± 7.86	0.38	14	0	0.939	0.907	-0.032	927
2_en_155.77	Distal	17.61 ± 7.09	155.77	11	0	0.870	0.826	-0.044	1073
3_en_111.84	Distal	26.16 ± 9.46	111.84	7	0	0.714	0.672	-0.041	328
4_en_69.2	Distal	22.60 ± 7.96	69.2	12	0.006	0.837	0.794	-0.042	840
4A_st_0.45	Distal	50.00	0.45	10	0	0.860	0.834	-0.027	813
5_en_62.17	Distal	50.00	62.17	11	0	0.933	0.868	-0.065	895
6_en_35.99	Distal	50.00	35.99	12	0	0.891	0.854	-0.037	840
7_en_39.18	Distal	45.58 ± 5.13	39.18	13	0.167	0.838	0.823	-0.015	579
8_en_27.41	Distal	22.98 ± 12.95	27.41	6	0.015	0.799	0.737	-0.062	1111

9_en_26.74	Distal	39.37 ± 8.12	26.74	10	0	0.889	0.824	-0.065	886
10_en_20.56	Distal	50.00	20.56	8	0	0.852	0.820	-0.033	842
11_st_0.14	Distal	35.51 ± 7.33	0.14	12	0	0.891	0.861	-0.030	951
13_st_0.37	Distal	50.00	0.37	8	0	0.739	0.727	-0.012	807
14_en_15.44	Distal	50.00	15.44	11	0	0.893	0.828	-0.066	721
15_st_0.88	Distal	50.00	0.88	8	0.073	0.851	0.829	-0.022	872
18_en_10.64	Distal	50.00	10.64	9	0.309	0.782	0.727	-0.056	68
19_en_11.22	Distal	50.00	11.22	9	0.238	0.745	0.803	0.058	673
20_en_15.24	Distal	50.00	15.24	6	0	0.680	0.634	-0.046	783
21_en_5.8	Distal	50.00	5.8	6	0.122	0.680	0.704	0.025	432
22_st_0.13	Distal	50.00	0.13	11	0	0.906	0.863	-0.043	799
23_en_6.19	Distal	23.38 ± 8.77	6.19	11	0.124	0.877	0.848	-0.029	821
24_st_0.41	Distal	50.00	0.41	11	0	0.858	0.789	-0.069	775
25_en_1.22	Distal	24.12 ± 7.76	1.22	10	0	0.908	0.832	-0.077	586
26_en_4.78	Distal	45.52 ± 5.57	4.78	9	0.004	0.853	0.840	-0.013	823
28_en_4.93	Distal		4.93	2	0	0.299	0.289	-0.010	66
Z_en_72.81	Distal	0	72.81	7	0.002	0.766	0.736	-0.030	249

Table S5 | Transmission ratios of microsatellite marker alleles depending on chromosomal side. Best linear unbiased estimates (BLUE) stem from a binomial GLMM with marker ID fitted as a random effect. Background rate is set as an offset parameter. Note that when testing with the background rate of 0.495, the BLUE is shifted, such that k = 0.5 at an observed transmission rate of 0.495. The actual observed transmission rate is estimated when the background rate is set to 0.5. CI = confidence interval.

**Data**	**Informative parent**	**Generation**	**Chromosomal side**	***N* transmissions (A, T)**	**BLUE**	**95% CI**	**P-value**	**Background rate**
All markers	Female	F1	Centromeric	2411, 2440	0.497	0.483–0.511	0.68	0.500
All markers	Female	BC1	Centromeric	2172, 2446	0.470	0.456–0.485	6×10^{-5}	0.500
All markers	Female	All	Centromeric	4583, 4886	0.484	0.474–0.494	0.0019	0.500
All markers	Male	F1	Centromeric	2693, 2679	0.501	0.484–0.518	0.91	0.500
All markers	Male	BC1	Centromeric	2154, 2233	0.491	0.476–0.506	0.24	0.500
All markers	Male	All	Centromeric	4847, 4912	0.496	0.483–0.510	0.60	0.500
All markers	Both combined	F1	Centromeric	5104, 5119	0.499	0.486–0.511	0.83	0.500
All markers	Both combined	BC1	Centromeric	4326, 4679	0.480	0.470–0.491	2×10^{-4}	0.500
All markers	Both combined	All	Centromeric	9430, 9798	0.491	0.481–0.500	0.053	0.500
All markers	Female	F1	Distal	2426, 2479	0.495	0.480–0.509	0.46	0.500
All markers	Female	BC1	Distal	2432, 2436	0.500	0.485–0.515	0.98	0.500
All markers	Female	All	Distal	4858, 4915	0.497	0.485–0.509	0.63	0.500
All markers	Male	F1	Distal	2631, 2719	0.492	0.478–0.506	0.24	0.500
All markers	Male	BC1	Distal	2072, 2118	0.495	0.479–0.510	0.48	0.500
All markers	Male	All	Distal	4703, 4837	0.494	0.482–0.506	0.32	0.500
All markers	Both combined	F1	Distal	5057, 5198	0.493	0.483–0.504	0.22	0.500
All markers	Both combined	BC1	Distal	4504, 4554	0.497	0.485–0.510	0.70	0.500
All markers	Both combined	All	Distal	9561, 9752	0.496	0.487–0.506	0.44	0.500
All markers	Female	F1	Both combined	4837, 4919	0.496	0.486–0.506	0.41	0.500
All markers	Female	BC1	Both combined	4604, 4882	0.486	0.474–0.497	0.011	0.500
All markers	Female	All	Both combined	9441, 9801	0.491	0.483–0.498	0.015	0.500
All markers	Male	F1	Both combined	5324, 5398	0.497	0.486–0.508	0.58	0.500
All markers	Male	BC1	Both combined	4226, 4351	0.493	0.482–0.503	0.18	0.500
All markers	Male	All	Both combined	9550, 9749	0.495	0.486–0.504	0.31	0.500
All markers	Both combined	F1	Both combined	10161, 10317	0.496	0.488–0.504	0.38	0.500
All markers	Both combined	BC1	Both combined	8830, 9233	0.489	0.480–0.497	0.011	0.500
All markers	Both combined	All	Both combined	18991, 19550	0.493	0.487–0.500	0.056	0.500
Heterozygous parents	Female	F1	Centromeric	1814, 1860	0.494	0.478–0.510	0.45	0.500
Heterozygous parents	Female	BC1	Centromeric	1435, 1655	0.464	0.447–0.482	8×10^{-5}	0.500

Heterozygous parents	Female	All	Centromeric	3249, 3515	0.480	0.468–0.492	0.0012	0.500
Heterozygous parents	Male	F1	Centromeric	1837, 1911	0.491	0.472–0.510	0.36	0.500
Heterozygous parents	Male	BC1	Centromeric	1385, 1342	0.507	0.485–0.530	0.52	0.500
Heterozygous parents	Male	All	Centromeric	3222, 3253	0.498	0.481–0.515	0.81	0.500
Heterozygous parents	Both combined	F1	Centromeric	3651, 3771	0.492	0.479–0.505	0.22	0.500
Heterozygous parents	Both combined	BC1	Centromeric	2820, 2997	0.485	0.469–0.501	0.058	0.500
Heterozygous parents	Both combined	All	Centromeric	6471, 6768	0.489	0.477–0.501	0.073	0.500
Heterozygous parents	Female	F1	Distal	1832, 1916	0.489	0.473–0.505	0.17	0.500
Heterozygous parents	Female	BC1	Distal	1394, 1429	0.494	0.475–0.512	0.51	0.500
Heterozygous parents	Female	All	Distal	3226, 3345	0.491	0.479–0.503	0.14	0.500
Heterozygous parents	Male	F1	Distal	1629, 1708	0.488	0.471–0.505	0.17	0.500
Heterozygous parents	Male	BC1	Distal	1276, 1262	0.503	0.483–0.522	0.78	0.500
Heterozygous parents	Male	All	Distal	2905, 2970	0.494	0.482–0.507	0.40	0.500
Heterozygous parents	Both combined	F1	Distal	3461, 3624	0.488	0.477–0.500	0.053	0.500
Heterozygous parents	Both combined	BC1	Distal	2670, 2691	0.498	0.484–0.512	0.79	0.500
Heterozygous parents	Both combined	All	Distal	6131, 6315	0.493	0.484–0.501	0.099	0.500
Heterozygous parents	Female	F1	Both combined	3646, 3776	0.491	0.480–0.503	0.13	0.500
Heterozygous parents	Female	BC1	Both combined	2829, 3084	0.478	0.466–0.491	9×10^{-4}	0.500
Heterozygous parents	Female	All	Both combined	6475, 6860	0.486	0.477–0.494	9×10^{-4}	0.500
Heterozygous parents	Male	F1	Both combined	3466, 3619	0.490	0.477–0.502	0.098	0.500
Heterozygous parents	Male	BC1	Both combined	2661, 2604	0.505	0.492–0.519	0.43	0.500
Heterozygous parents	Male	All	Both combined	6127, 6223	0.496	0.486–0.506	0.44	0.500
Heterozygous parents	Both combined	F1	Both combined	7112, 7395	0.490	0.482–0.499	0.023	0.500
Heterozygous parents	Both combined	BC1	Both combined	5490, 5688	0.491	0.481–0.502	0.11	0.500
Heterozygous parents	Both combined	All	Both combined	12602, 13083	0.491	0.483–0.498	0.014	0.500
No inbreeding	Female	F1	Centromeric	1400, 1409	0.499	0.477–0.520	0.89	0.500
No inbreeding	Female	BC1	Centromeric	830, 906	0.478	0.454–0.502	0.078	0.500
No inbreeding	Female	All	Centromeric	2230, 2315	0.491	0.475–0.507	0.26	0.500
No inbreeding	Male	F1	Centromeric	1553, 1526	0.501	0.477–0.525	0.91	0.500
No inbreeding	Male	BC1	Centromeric	807, 818	0.497	0.472–0.521	0.78	0.500
No inbreeding	Male	All	Centromeric	2360, 2344	0.501	0.483–0.519	0.93	0.500
No inbreeding	Both combined	F1	Centromeric	2953, 2935	0.500	0.480–0.519	0.96	0.500
No inbreeding	Both combined	BC1	Centromeric	1637, 1724	0.487	0.470–0.504	0.13	0.500
No inbreeding	Both combined	All	Centromeric	4590, 4659	0.497	0.484–0.510	0.61	0.500
No inbreeding	Female	F1	Distal	1348, 1373	0.495	0.476–0.514	0.63	0.500
No inbreeding	Female	BC1	Distal	948, 914	0.509	0.486–0.532	0.43	0.500
No inbreeding	Female	All	Distal	2296, 2287	0.501	0.487–0.515	0.89	0.500

No inbreeding	Male	F1	Distal	1567, 1666	0.485	0.467–0.502	0.082	0.500
No inbreeding	Male	BC1	Distal	825, 824	0.500	0.476–0.524	0.98	0.500
No inbreeding	Male	All	Distal	2392, 2490	0.490	0.476–0.504	0.16	0.500
No inbreeding	Both combined	F1	Distal	2915, 3039	0.490	0.477–0.502	0.11	0.500
No inbreeding	Both combined	BC1	Distal	1773, 1738	0.505	0.488–0.522	0.55	0.500
No inbreeding	Both combined	All	Distal	4688, 4777	0.495	0.485–0.505	0.36	0.500
No inbreeding	Female	F1	Both combined	2748, 2782	0.497	0.483–0.511	0.66	0.500
No inbreeding	Female	BC1	Both combined	1778, 1820	0.494	0.478–0.511	0.48	0.500
No inbreeding	Female	All	Both combined	4526, 4602	0.496	0.485–0.507	0.46	0.500
No inbreeding	Male	F1	Both combined	3120, 3192	0.494	0.479–0.508	0.38	0.500
No inbreeding	Male	BC1	Both combined	1632, 1642	0.498	0.481–0.516	0.86	0.500
No inbreeding	Male	All	Both combined	4752, 4834	0.495	0.484–0.507	0.42	0.500
No inbreeding	Both combined	F1	Both combined	5868, 5974	0.495	0.484–0.506	0.36	0.500
No inbreeding	Both combined	BC1	Both combined	3410, 3462	0.496	0.484–0.508	0.53	0.500
No inbreeding	Both combined	All	Both combined	9278, 9436	0.496	0.488–0.503	0.27	0.500
All markers	Female	F1	Centromeric	2411, 2440	0.502	0.488–0.516	0.76	0.495
All markers	Female	BC1	Centromeric	2172, 2446	0.475	0.461–0.490	9×10^{-4}	0.495
All markers	Female	All	Centromeric	4583, 4886	0.489	0.479–0.499	0.035	0.495
All markers	Male	F1	Centromeric	2693, 2679	0.506	0.489–0.523	0.47	0.495
All markers	Male	BC1	Centromeric	2154, 2233	0.496	0.481–0.511	0.61	0.495
All markers	Male	All	Centromeric	4847, 4912	0.502	0.488–0.515	0.81	0.495
All markers	Both combined	F1	Centromeric	5104, 5119	0.504	0.491–0.516	0.55	0.495
All markers	Both combined	BC1	Centromeric	4326, 4679	0.486	0.475–0.496	0.0062	0.495
All markers	Both combined	All	Centromeric	9430, 9798	0.496	0.486–0.505	0.38	0.495
All markers	Female	F1	Distal	2426, 2479	0.500	0.485–0.514	0.97	0.495
All markers	Female	BC1	Distal	2432, 2436	0.505	0.490–0.520	0.52	0.495
All markers	Female	All	Distal	4858, 4915	0.502	0.490–0.514	0.71	0.495
All markers	Male	F1	Distal	2631, 2719	0.497	0.483–0.511	0.67	0.495
All markers	Male	BC1	Distal	2072, 2118	0.500	0.484–0.515	0.97	0.495
All markers	Male	All	Distal	4703, 4837	0.499	0.487–0.511	0.87	0.495
All markers	Both combined	F1	Distal	5057, 5198	0.499	0.488–0.509	0.79	0.495
All markers	Both combined	BC1	Distal	4504, 4554	0.503	0.490–0.516	0.69	0.495
All markers	Both combined	All	Distal	9561, 9752	0.501	0.492–0.511	0.79	0.495
All markers	Female	F1	Both combined	4837, 4919	0.501	0.491–0.511	0.85	0.495
All markers	Female	BC1	Both combined	4604, 4882	0.491	0.480–0.502	0.10	0.495
All markers	Female	All	Both combined	9441, 9801	0.496	0.488–0.503	0.28	0.495
All markers	Male	F1	Both combined	5324, 5398	0.502	0.491–0.513	0.71	0.495

All markers	Male	BC1	Both combined	4226, 4351	0.498	0.487–0.509	0.69	0.495
All markers	Male	All	Both combined	9550, 9749	0.500	0.491–0.510	0.92	0.495
All markers	Both combined	F1	Both combined	10161, 10317	0.501	0.493–0.510	0.73	0.495
All markers	Both combined	BC1	Both combined	8830, 9233	0.494	0.486–0.503	0.17	0.495
All markers	Both combined	All	Both combined	18991, 19550	0.499	0.492–0.505	0.67	0.495
Heterozygous parents	Female	F1	Centromeric	1814, 1860	0.499	0.483–0.515	0.89	0.495
Heterozygous parents	Female	BC1	Centromeric	1435, 1655	0.470	0.452–0.487	7×10^{-4}	0.495
Heterozygous parents	Female	All	Centromeric	3249, 3515	0.485	0.474–0.497	0.017	0.495
Heterozygous parents	Male	F1	Centromeric	1837, 1911	0.496	0.477–0.515	0.70	0.495
Heterozygous parents	Male	BC1	Centromeric	1385, 1342	0.513	0.490–0.535	0.27	0.495
Heterozygous parents	Male	All	Centromeric	3222, 3253	0.503	0.486–0.520	0.73	0.495
Heterozygous parents	Both combined	F1	Centromeric	3651, 3771	0.497	0.485–0.510	0.66	0.495
Heterozygous parents	Both combined	BC1	Centromeric	2820, 2997	0.490	0.474–0.506	0.21	0.495
Heterozygous parents	Both combined	All	Centromeric	6471, 6768	0.494	0.482–0.506	0.34	0.495
Heterozygous parents	Female	F1	Distal	1832, 1916	0.494	0.478–0.510	0.46	0.495
Heterozygous parents	Female	BC1	Distal	1394, 1429	0.499	0.481–0.517	0.91	0.495
Heterozygous parents	Female	All	Distal	3226, 3345	0.496	0.484–0.508	0.53	0.495
Heterozygous parents	Male	F1	Distal	1629, 1708	0.493	0.476–0.510	0.44	0.495
Heterozygous parents	Male	BC1	Distal	1276, 1262	0.508	0.488–0.527	0.43	0.495
Heterozygous parents	Male	All	Distal	2905, 2970	0.500	0.487–0.512	0.95	0.495
Heterozygous parents	Both combined	F1	Distal	3461, 3624	0.494	0.482–0.505	0.29	0.495
Heterozygous parents	Both combined	BC1	Distal	2670, 2691	0.503	0.489–0.517	0.64	0.495
Heterozygous parents	Both combined	All	Distal	6131, 6315	0.498	0.489–0.507	0.62	0.495
Heterozygous parents	Female	F1	Both combined	3646, 3776	0.496	0.485–0.508	0.53	0.495
Heterozygous parents	Female	BC1	Both combined	2829, 3084	0.484	0.471–0.496	0.012	0.495
Heterozygous parents	Female	All	Both combined	6475, 6860	0.491	0.482–0.499	0.032	0.495
Heterozygous parents	Male	F1	Both combined	3466, 3619	0.495	0.482–0.507	0.40	0.495
Heterozygous parents	Male	BC1	Both combined	2661, 2604	0.511	0.497–0.524	0.13	0.495
Heterozygous parents	Male	All	Both combined	6127, 6223	0.501	0.491–0.511	0.84	0.495
Heterozygous parents	Both combined	F1	Both combined	7112, 7395	0.495	0.487–0.504	0.29	0.495
Heterozygous parents	Both combined	BC1	Both combined	5490, 5688	0.496	0.486–0.507	0.52	0.495
Heterozygous parents	Both combined	All	Both combined	12602, 13083	0.496	0.489–0.503	0.27	0.495
No inbreeding	Female	F1	Centromeric	1400, 1409	0.504	0.482–0.525	0.73	0.495
No inbreeding	Female	BC1	Centromeric	830, 906	0.483	0.459–0.508	0.18	0.495
No inbreeding	Female	All	Centromeric	2230, 2315	0.496	0.480–0.512	0.63	0.495
No inbreeding	Male	F1	Centromeric	1553, 1526	0.507	0.483–0.530	0.59	0.495
No inbreeding	Male	BC1	Centromeric	807, 818	0.502	0.477–0.526	0.89	0.495

No inbreeding	Male	All	Centromeric	2360, 2344	0.506	0.488–0.524	0.51	0.495
No inbreeding	Both combined	F1	Centromeric	2953, 2935	0.505	0.485–0.524	0.63	0.495
No inbreeding	Both combined	BC1	Centromeric	1637, 1724	0.492	0.475–0.509	0.37	0.495
No inbreeding	Both combined	All	Centromeric	4590, 4659	0.502	0.489–0.515	0.79	0.495
No inbreeding	Female	F1	Distal	1348, 1373	0.501	0.481–0.520	0.96	0.495
No inbreeding	Female	BC1	Distal	948, 914	0.514	0.492–0.537	0.22	0.495
No inbreeding	Female	All	Distal	2296, 2287	0.506	0.492–0.521	0.41	0.495
No inbreeding	Male	F1	Distal	1567, 1666	0.490	0.473–0.507	0.25	0.495
No inbreeding	Male	BC1	Distal	825, 824	0.505	0.481–0.530	0.66	0.495
No inbreeding	Male	All	Distal	2392, 2490	0.495	0.481–0.509	0.50	0.495
No inbreeding	Both combined	F1	Distal	2915, 3039	0.495	0.482–0.507	0.42	0.495
No inbreeding	Both combined	BC1	Distal	1773, 1738	0.510	0.494–0.527	0.23	0.495
No inbreeding	Both combined	All	Distal	4688, 4777	0.500	0.490–0.511	0.93	0.495
No inbreeding	Female	F1	Both combined	2748, 2782	0.502	0.488–0.516	0.78	0.495
No inbreeding	Female	BC1	Both combined	1778, 1820	0.499	0.483–0.516	0.93	0.495
No inbreeding	Female	All	Both combined	4526, 4602	0.501	0.490–0.512	0.84	0.495
No inbreeding	Male	F1	Both combined	3120, 3192	0.499	0.485–0.513	0.87	0.495
No inbreeding	Male	BC1	Both combined	1632, 1642	0.504	0.487–0.521	0.68	0.495
No inbreeding	Male	All	Both combined	4752, 4834	0.501	0.489–0.512	0.93	0.495
No inbreeding	Both combined	F1	Both combined	5868, 5974	0.500	0.489–0.511	0.98	0.495
No inbreeding	Both combined	BC1	Both combined	3410, 3462	0.501	0.490–0.513	0.82	0.495
No inbreeding	Both combined	All	Both combined	9278, 9436	0.501	0.493–0.508	0.80	0.495

Table S6 | Transmission ratios of all microsatellite marker alleles against a background transmission rate of 0.495. Best linear unbiased estimates (BLUE) stem from a binomial GLM with offset parameter 0.495.

Informative parent	Generation	Chromosomal side	Microsatellite marker	*N* trans-missions (A, T)	BLUE	95% confidence interval	P-value
Female	F1	Centromeric	1_cen_98.17	110, 107	0.512	0.446–0.578	0.72
Female	F1	Centromeric	1A_cen_62.53	118, 99	0.549	0.482–0.614	0.15
Female	F1	Centromeric	2_cen_76.29	117, 100	0.544	0.478–0.610	0.19
Female	F1	Centromeric	3_cen_40.34	114, 103	0.530	0.464–0.596	0.37
Female	F1	Centromeric	4_cen_16.82	116, 101	0.540	0.473–0.605	0.24
Female	F1	Centromeric	4A_en_19.79	103, 108	0.493	0.426–0.561	0.85
Female	F1	Centromeric	5rand_cen_0.26	44, 76	0.371	0.288–0.460	0.0055
Female	F1	Centromeric	6_cen_0.89	102, 114	0.477	0.411–0.544	0.51
Female	F1	Centromeric	8_cen_1.38	111, 106	0.517	0.450–0.583	0.62
Female	F1	Centromeric	9_st_0.96	119, 98	0.553	0.487–0.619	0.12
Female	F1	Centromeric	10_st_0.86	102, 104	0.500	0.432–0.568	0.99
Female	F1	Centromeric	12_st_0.77	64, 78	0.456	0.375–0.538	0.29
Female	F1	Centromeric	15_en_13.76	104, 113	0.484	0.418–0.551	0.65
Female	F1	Centromeric	17_st_0.7	102, 115	0.475	0.409–0.542	0.47
Female	F1	Centromeric	18_st_0.48	100, 117	0.466	0.400–0.532	0.32
Female	F1	Centromeric	19_st_0.97	108, 109	0.503	0.437–0.569	0.93
Female	F1	Centromeric	21_st_0.22	107, 110	0.498	0.432–0.564	0.96
Female	F1	Centromeric	22_en_3.1	107, 110	0.498	0.432–0.564	0.96
Female	F1	Centromeric	23_st_0.68	107, 110	0.498	0.432–0.564	0.96
Female	F1	Centromeric	25_st_0.03	110, 107	0.512	0.446–0.578	0.72
Female	F1	Centromeric	28_st_0.29	110, 107	0.512	0.446–0.578	0.72
Female	F1	Centromeric	24_en_7.89	84, 87	0.496	0.422–0.571	0.92
Female	F1	Centromeric	7_cen_4.65	47, 50	0.490	0.391–0.588	0.84
Female	F1	Centromeric	11_en_20.8	10, 12	0.460	0.264–0.664	0.71
Female	F1	Centromeric	13_en_16.75	10, 12	0.460	0.264–0.664	0.71
Female	F1	Centromeric	20_st_1.91	50, 47	0.521	0.422–0.618	0.68
Female	F1	Centromeric	26_st_0.2	35, 40	0.472	0.361–0.584	0.63
Female	BC1	Centromeric	1_cen_98.17	83, 99	0.461	0.390–0.534	0.30
Female	BC1	Centromeric	4A_en_19.79	41, 37	0.531	0.420–0.639	0.59
Female	BC1	Centromeric	5rand_cen_0.26	62, 50	0.559	0.466–0.648	0.21
Female	BC1	Centromeric	8_cen_1.38	90, 107	0.462	0.393–0.532	0.29
Female	BC1	Centromeric	12_st_0.77	84, 89	0.491	0.417–0.565	0.81
Female	BC1	Centromeric	15_en_13.76	128, 149	0.467	0.409–0.526	0.28
Female	BC1	Centromeric	17_st_0.7	86, 104	0.458	0.388–0.529	0.25
Female	BC1	Centromeric	19_st_0.97	129, 122	0.519	0.457–0.580	0.55
Female	BC1	Centromeric	21_st_0.22	65, 55	0.547	0.457–0.634	0.31
Female	BC1	Centromeric	23_st_0.68	132, 159	0.459	0.402–0.516	0.16
Female	BC1	Centromeric	1A_cen_62.53	98, 116	0.463	0.397–0.530	0.28
Female	BC1	Centromeric	4_cen_16.82	105, 105	0.505	0.438–0.572	0.88
Female	BC1	Centromeric	6_cen_0.89	108, 146	0.430	0.370–0.492	0.027
Female	BC1	Centromeric	7_cen_4.65	87, 95	0.483	0.411–0.556	0.65
Female	BC1	Centromeric	10_st_0.86	107, 126	0.464	0.401–0.529	0.28
Female	BC1	Centromeric	18_st_0.48	98, 123	0.449	0.384–0.514	0.13
Female	BC1	Centromeric	24_en_7.89	45, 53	0.464	0.367–0.563	0.48
Female	BC1	Centromeric	28_st_0.29	103, 146	0.419	0.358–0.481	0.011
Female	BC1	Centromeric	2_cen_76.29	101, 105	0.495	0.428–0.563	0.90
Female	BC1	Centromeric	3_cen_40.34	52, 73	0.421	0.337–0.509	0.079
Female	BC1	Centromeric	25_st_0.03	97, 85	0.538	0.466–0.610	0.30
Female	BC1	Centromeric	9_st_0.96	76, 97	0.444	0.371–0.519	0.14
Female	BC1	Centromeric	11_en_20.8	53, 56	0.491	0.398–0.585	0.86
Female	BC1	Centromeric	22_en_3.1	77, 86	0.478	0.402–0.554	0.57

Female	BC1	Centromeric	13_en_16.75	45, 42	0.522	0.418–0.625	0.68
Female	BC1	Centromeric	20_st_1.91	20, 21	0.493	0.344–0.643	0.93
Female	All	Centromeric	1_cen_98.17	193, 206	0.489	0.440–0.538	0.66
Female	All	Centromeric	1A_cen_62.53	216, 215	0.506	0.459–0.553	0.79
Female	All	Centromeric	2_cen_76.29	218, 205	0.521	0.473–0.568	0.40
Female	All	Centromeric	3_cen_40.34	166, 176	0.491	0.438–0.543	0.73
Female	All	Centromeric	4_cen_16.82	221, 206	0.523	0.475–0.570	0.35
Female	All	Centromeric	4A_en_19.79	144, 145	0.503	0.446–0.561	0.91
Female	All	Centromeric	5rand_cen_0.26	106, 126	0.462	0.398–0.526	0.25
Female	All	Centromeric	6_cen_0.89	210, 260	0.452	0.407–0.497	0.038
Female	All	Centromeric	8_cen_1.38	201, 213	0.491	0.443–0.539	0.70
Female	All	Centromeric	9_st_0.96	195, 195	0.505	0.456–0.555	0.84
Female	All	Centromeric	10_st_0.86	209, 230	0.481	0.435–0.528	0.43
Female	All	Centromeric	12_st_0.77	148, 167	0.475	0.420–0.530	0.37
Female	All	Centromeric	15_en_13.76	232, 262	0.475	0.431–0.519	0.26
Female	All	Centromeric	17_st_0.7	188, 219	0.467	0.419–0.516	0.18
Female	All	Centromeric	18_st_0.48	198, 240	0.457	0.411–0.504	0.074
Female	All	Centromeric	19_st_0.97	237, 231	0.512	0.466–0.557	0.62
Female	All	Centromeric	21_st_0.22	172, 165	0.516	0.462–0.569	0.57
Female	All	Centromeric	22_en_3.1	184, 196	0.489	0.439–0.540	0.68
Female	All	Centromeric	23_st_0.68	239, 269	0.476	0.432–0.519	0.27
Female	All	Centromeric	25_st_0.03	207, 192	0.524	0.475–0.573	0.34
Female	All	Centromeric	28_st_0.29	213, 253	0.462	0.417–0.508	0.10
Female	All	Centromeric	24_en_7.89	129, 140	0.485	0.425–0.544	0.62
Female	All	Centromeric	7_cen_4.65	134, 145	0.485	0.427–0.544	0.63
Female	All	Centromeric	11_en_20.8	63, 68	0.486	0.401–0.571	0.75
Female	All	Centromeric	13_en_16.75	55, 54	0.510	0.416–0.602	0.84
Female	All	Centromeric	20_st_1.91	70, 68	0.512	0.429–0.595	0.77
Female	All	Centromeric	26_st_0.2	35, 40	0.472	0.361–0.584	0.63
Male	F1	Centromeric	1_cen_98.17	101, 123	0.456	0.391–0.521	0.19
Male	F1	Centromeric	1A_cen_62.53	111, 112	0.503	0.438–0.568	0.93
Male	F1	Centromeric	2_cen_76.29	116, 108	0.523	0.458–0.588	0.49
Male	F1	Centromeric	3_cen_40.34	126, 98	0.568	0.502–0.631	0.043
Male	F1	Centromeric	4_cen_16.82	126, 97	0.570	0.505–0.634	0.037
Male	F1	Centromeric	4A_en_19.79	98, 89	0.529	0.458–0.600	0.42
Male	F1	Centromeric	6_cen_0.89	107, 116	0.485	0.420–0.550	0.65
Male	F1	Centromeric	7_cen_4.65	113, 111	0.510	0.444–0.575	0.77
Male	F1	Centromeric	8_cen_1.38	113, 111	0.510	0.444–0.575	0.77
Male	F1	Centromeric	9_st_0.96	117, 107	0.527	0.462–0.592	0.41
Male	F1	Centromeric	10_st_0.86	107, 101	0.520	0.452–0.587	0.57
Male	F1	Centromeric	11_en_20.8	71, 80	0.475	0.397–0.555	0.54
Male	F1	Centromeric	12_st_0.77	104, 118	0.474	0.408–0.539	0.43
Male	F1	Centromeric	15_en_13.76	97, 127	0.438	0.374–0.504	0.065
Male	F1	Centromeric	17_st_0.7	107, 117	0.483	0.418–0.548	0.61
Male	F1	Centromeric	18_st_0.48	112, 112	0.505	0.440–0.570	0.88
Male	F1	Centromeric	19_st_0.97	105, 118	0.476	0.411–0.542	0.47
Male	F1	Centromeric	20_st_1.91	36, 41	0.473	0.363–0.584	0.63
Male	F1	Centromeric	21_st_0.22	128, 95	0.579	0.514–0.643	0.019
Male	F1	Centromeric	22_en_3.1	105, 119	0.474	0.409–0.539	0.43
Male	F1	Centromeric	23_st_0.68	97, 126	0.440	0.376–0.506	0.074
Male	F1	Centromeric	25_st_0.03	125, 99	0.563	0.498–0.627	0.059
Male	F1	Centromeric	28_st_0.29	103, 120	0.467	0.402–0.533	0.33
Male	F1	Centromeric	Z_cen_27.51	127, 96	0.575	0.509–0.638	0.026
Male	F1	Centromeric	5rand_cen_0.26	30, 41	0.428	0.316–0.544	0.22
Male	F1	Centromeric	24_en_7.89	63, 45	0.588	0.494–0.678	0.067
Male	F1	Centromeric	26_st_0.2	12, 17	0.419	0.251–0.600	0.38
Male	F1	Centromeric	13_en_16.75	36, 35	0.512	0.397–0.626	0.84
Male	BC1	Centromeric	1_cen_98.17	86, 99	0.470	0.399–0.542	0.42

Male	BC1	Centromeric	1A_cen_62.53	148, 143	0.514	0.456–0.571	0.64
Male	BC1	Centromeric	2_cen_76.29	167, 164	0.510	0.456–0.563	0.72
Male	BC1	Centromeric	6_cen_0.89	77, 59	0.571	0.487–0.652	0.10
Male	BC1	Centromeric	15_en_13.76	158, 146	0.525	0.469–0.581	0.39
Male	BC1	Centromeric	17_st_0.7	106, 117	0.480	0.415–0.546	0.56
Male	BC1	Centromeric	21_st_0.22	100, 90	0.531	0.460–0.602	0.39
Male	BC1	Centromeric	22_en_3.1	109, 121	0.479	0.415–0.544	0.53
Male	BC1	Centromeric	3_cen_40.34	83, 62	0.577	0.496–0.656	0.063
Male	BC1	Centromeric	4_cen_16.82	53, 62	0.466	0.376–0.557	0.47
Male	BC1	Centromeric	8_cen_1.38	51, 78	0.400	0.318–0.486	0.025
Male	BC1	Centromeric	9_st_0.96	142, 159	0.477	0.421–0.533	0.42
Male	BC1	Centromeric	10_st_0.86	74, 95	0.443	0.369–0.518	0.14
Male	BC1	Centromeric	18_st_0.48	84, 88	0.494	0.419–0.568	0.87
Male	BC1	Centromeric	19_st_0.97	101, 99	0.510	0.441–0.579	0.77
Male	BC1	Centromeric	20_st_1.91	47, 49	0.495	0.396–0.594	0.92
Male	BC1	Centromeric	24_en_7.89	33, 31	0.521	0.399–0.640	0.74
Male	BC1	Centromeric	25_st_0.03	119, 105	0.536	0.471–0.601	0.28
Male	BC1	Centromeric	28_st_0.29	125, 136	0.484	0.424–0.545	0.61
Male	BC1	Centromeric	5rand_cen_0.26	32, 58	0.360	0.266–0.463	0.0091
Male	BC1	Centromeric	12_st_0.77	29, 37	0.444	0.328–0.565	0.37
Male	BC1	Centromeric	11_en_20.8	28, 36	0.443	0.325–0.565	0.36
Male	BC1	Centromeric	23_st_0.68	116, 116	0.505	0.441–0.569	0.88
Male	BC1	Centromeric	4A_en_19.79	52, 38	0.583	0.480–0.681	0.12
Male	BC1	Centromeric	13_en_16.75	28, 38	0.429	0.314–0.550	0.25
Male	BC1	Centromeric	7_cen_4.65	4, 5	0.450	0.168–0.755	0.76
Male	BC1	Centromeric	Z_cen_27.51	2, 2	0.505	0.109–0.895	0.98
Male	All	Centromeric	1_cen_98.17	187, 222	0.462	0.414–0.511	0.13
Male	All	Centromeric	1A_cen_62.53	259, 255	0.509	0.466–0.552	0.68
Male	All	Centromeric	2_cen_76.29	283, 272	0.515	0.473–0.556	0.48
Male	All	Centromeric	3_cen_40.34	209, 160	0.571	0.521–0.621	0.0062
Male	All	Centromeric	4_cen_16.82	179, 159	0.535	0.481–0.587	0.20
Male	All	Centromeric	4A_en_19.79	150, 127	0.547	0.488–0.605	0.12
Male	All	Centromeric	6_cen_0.89	184, 175	0.518	0.466–0.569	0.50
Male	All	Centromeric	7_cen_4.65	117, 116	0.507	0.443–0.571	0.82
Male	All	Centromeric	8_cen_1.38	164, 189	0.470	0.418–0.522	0.26
Male	All	Centromeric	9_st_0.96	259, 266	0.498	0.456–0.541	0.94
Male	All	Centromeric	10_st_0.86	181, 196	0.485	0.435–0.536	0.57
Male	All	Centromeric	11_en_20.8	99, 116	0.466	0.400–0.532	0.31
Male	All	Centromeric	12_st_0.77	133, 155	0.467	0.410–0.525	0.26
Male	All	Centromeric	15_en_13.76	255, 273	0.488	0.446–0.531	0.58
Male	All	Centromeric	17_st_0.7	213, 234	0.482	0.436–0.528	0.44
Male	All	Centromeric	18_st_0.48	196, 200	0.500	0.451–0.549	1.00
Male	All	Centromeric	19_st_0.97	206, 217	0.492	0.445–0.540	0.75
Male	All	Centromeric	20_st_1.91	83, 90	0.485	0.411–0.559	0.69
Male	All	Centromeric	21_st_0.22	228, 185	0.557	0.509–0.605	0.020
Male	All	Centromeric	22_en_3.1	214, 240	0.477	0.431–0.522	0.32
Male	All	Centromeric	23_st_0.68	213, 242	0.473	0.428–0.519	0.25
Male	All	Centromeric	25_st_0.03	244, 204	0.550	0.504–0.595	0.035
Male	All	Centromeric	28_st_0.29	228, 256	0.476	0.432–0.521	0.30
Male	All	Centromeric	Z_cen_27.51	129, 98	0.573	0.509–0.636	0.027
Male	All	Centromeric	5rand_cen_0.26	62, 99	0.390	0.317–0.467	0.0057
Male	All	Centromeric	24_en_7.89	96, 76	0.563	0.489–0.636	0.10
Male	All	Centromeric	26_st_0.2	12, 17	0.419	0.251–0.600	0.38
Male	All	Centromeric	13_en_16.75	64, 73	0.472	0.390–0.556	0.52
Both combined	F1	Centromeric	1_cen_98.17	211, 230	0.484	0.437–0.530	0.49
Both combined	F1	Centromeric	1A_cen_62.53	229, 211	0.526	0.479–0.572	0.28
Both combined	F1	Centromeric	2_cen_76.29	233, 208	0.533	0.487–0.580	0.16
Both combined	F1	Centromeric	3_cen_40.34	240, 201	0.549	0.503–0.595	0.038

Both combined	F1	Centromeric	4_cen_16.82	242, 198	0.555	0.508–0.601	0.021
Both combined	F1	Centromeric	4A_en_19.79	201, 197	0.510	0.461–0.559	0.68
Both combined	F1	Centromeric	6_cen_0.89	209, 230	0.481	0.435–0.528	0.43
Both combined	F1	Centromeric	7_cen_4.65	160, 161	0.504	0.449–0.558	0.90
Both combined	F1	Centromeric	8_cen_1.38	224, 217	0.513	0.466–0.560	0.58
Both combined	F1	Centromeric	9_st_0.96	236, 205	0.540	0.494–0.586	0.091
Both combined	F1	Centromeric	10_st_0.86	209, 205	0.510	0.462–0.558	0.68
Both combined	F1	Centromeric	11_en_20.8	81, 92	0.473	0.400–0.548	0.48
Both combined	F1	Centromeric	12_st_0.77	168, 196	0.467	0.416–0.518	0.20
Both combined	F1	Centromeric	15_en_13.76	201, 240	0.461	0.415–0.508	0.10
Both combined	F1	Centromeric	17_st_0.7	209, 232	0.479	0.433–0.526	0.38
Both combined	F1	Centromeric	18_st_0.48	212, 229	0.486	0.439–0.533	0.55
Both combined	F1	Centromeric	19_st_0.97	213, 227	0.489	0.443–0.536	0.65
Both combined	F1	Centromeric	20_st_1.91	86, 88	0.499	0.425–0.573	0.99
Both combined	F1	Centromeric	21_st_0.22	235, 205	0.539	0.493–0.585	0.10
Both combined	F1	Centromeric	22_en_3.1	212, 229	0.486	0.439–0.533	0.55
Both combined	F1	Centromeric	23_st_0.68	204, 236	0.469	0.422–0.515	0.19
Both combined	F1	Centromeric	25_st_0.03	235, 206	0.538	0.491–0.584	0.11
Both combined	F1	Centromeric	28_st_0.29	213, 227	0.489	0.443–0.536	0.65
Both combined	F1	Centromeric	Z_cen_27.51	127, 96	0.575	0.509–0.638	0.026
Both combined	F1	Centromeric	5rand_cen_0.26	74, 117	0.392	0.325–0.463	0.0032
Both combined	F1	Centromeric	24_en_7.89	147, 132	0.532	0.473–0.590	0.28
Both combined	F1	Centromeric	13_en_16.75	46, 47	0.500	0.399–0.600	1.00
Both combined	F1	Centromeric	26_st_0.2	47, 57	0.457	0.363–0.553	0.38
Both combined	BC1	Centromeric	1_cen_98.17	169, 198	0.466	0.415–0.517	0.19
Both combined	BC1	Centromeric	4A_en_19.79	93, 75	0.559	0.483–0.632	0.13
Both combined	BC1	Centromeric	5rand_cen_0.26	94, 108	0.470	0.402–0.539	0.40
Both combined	BC1	Centromeric	8_cen_1.38	141, 185	0.438	0.384–0.492	0.025
Both combined	BC1	Centromeric	12_st_0.77	113, 126	0.478	0.415–0.541	0.50
Both combined	BC1	Centromeric	15_en_13.76	286, 295	0.497	0.457–0.538	0.90
Both combined	BC1	Centromeric	17_st_0.7	192, 221	0.470	0.422–0.518	0.22
Both combined	BC1	Centromeric	19_st_0.97	230, 221	0.515	0.469–0.561	0.52
Both combined	BC1	Centromeric	21_st_0.22	165, 145	0.537	0.482–0.592	0.19
Both combined	BC1	Centromeric	23_st_0.68	248, 275	0.479	0.437–0.522	0.34
Both combined	BC1	Centromeric	1A_cen_62.53	246, 259	0.492	0.449–0.536	0.73
Both combined	BC1	Centromeric	4_cen_16.82	158, 167	0.491	0.437–0.546	0.75
Both combined	BC1	Centromeric	6_cen_0.89	185, 205	0.480	0.430–0.529	0.42
Both combined	BC1	Centromeric	7_cen_4.65	91, 100	0.482	0.411–0.552	0.61
Both combined	BC1	Centromeric	10_st_0.86	181, 221	0.455	0.407–0.504	0.074
Both combined	BC1	Centromeric	18_st_0.48	182, 211	0.468	0.419–0.518	0.21
Both combined	BC1	Centromeric	24_en_7.89	78, 84	0.487	0.410–0.563	0.73
Both combined	BC1	Centromeric	28_st_0.29	228, 282	0.452	0.409–0.496	0.031
Both combined	BC1	Centromeric	2_cen_76.29	268, 269	0.504	0.462–0.546	0.84
Both combined	BC1	Centromeric	3_cen_40.34	135, 135	0.505	0.446–0.565	0.87
Both combined	BC1	Centromeric	25_st_0.03	216, 190	0.537	0.489–0.585	0.13
Both combined	BC1	Centromeric	9_st_0.96	218, 256	0.465	0.420–0.510	0.13
Both combined	BC1	Centromeric	11_en_20.8	81, 92	0.473	0.400–0.548	0.48
Both combined	BC1	Centromeric	22_en_3.1	186, 207	0.478	0.429–0.528	0.39
Both combined	BC1	Centromeric	13_en_16.75	73, 80	0.482	0.404–0.561	0.66
Both combined	BC1	Centromeric	20_st_1.91	67, 70	0.494	0.411–0.577	0.89
Both combined	BC1	Centromeric	Z_cen_27.51	2, 2	0.505	0.109–0.895	0.98
Both combined	All	Centromeric	1_cen_98.17	380, 428	0.475	0.441–0.510	0.16
Both combined	All	Centromeric	1A_cen_62.53	475, 470	0.508	0.476–0.540	0.63
Both combined	All	Centromeric	2_cen_76.29	501, 477	0.517	0.486–0.549	0.28
Both combined	All	Centromeric	3_cen_40.34	375, 336	0.533	0.496–0.569	0.082
Both combined	All	Centromeric	4_cen_16.82	400, 365	0.528	0.493–0.563	0.12
Both combined	All	Centromeric	4A_en_19.79	294, 272	0.525	0.483–0.566	0.24
Both combined	All	Centromeric	6_cen_0.89	394, 435	0.480	0.446–0.514	0.26

Both combined	All	Centromeric	7_cen_4.65	251, 261	0.495	0.452–0.539	0.83
Both combined	All	Centromeric	8_cen_1.38	365, 402	0.481	0.446–0.516	0.29
Both combined	All	Centromeric	9_st_0.96	454, 461	0.501	0.469–0.534	0.94
Both combined	All	Centromeric	10_st_0.86	390, 426	0.483	0.449–0.517	0.33
Both combined	All	Centromeric	11_en_20.8	162, 184	0.473	0.421–0.526	0.32
Both combined	All	Centromeric	12_st_0.77	281, 322	0.471	0.431–0.511	0.16
Both combined	All	Centromeric	15_en_13.76	487, 535	0.482	0.451–0.512	0.24
Both combined	All	Centromeric	17_st_0.7	401, 453	0.475	0.441–0.508	0.14
Both combined	All	Centromeric	18_st_0.48	394, 440	0.478	0.444–0.512	0.20
Both combined	All	Centromeric	19_st_0.97	443, 448	0.502	0.470–0.535	0.89
Both combined	All	Centromeric	20_st_1.91	153, 158	0.497	0.442–0.553	0.92
Both combined	All	Centromeric	21_st_0.22	400, 350	0.538	0.503–0.574	0.035
Both combined	All	Centromeric	22_en_3.1	398, 436	0.482	0.449–0.516	0.31
Both combined	All	Centromeric	23_st_0.68	452, 511	0.475	0.443–0.506	0.11
Both combined	All	Centromeric	25_st_0.03	451, 396	0.538	0.504–0.571	0.029
Both combined	All	Centromeric	28_st_0.29	441, 509	0.469	0.438–0.501	0.059
Both combined	All	Centromeric	Z_cen_27.51	129, 98	0.573	0.509–0.636	0.027
Both combined	All	Centromeric	5rand_cen_0.26	168, 225	0.433	0.384–0.482	0.0078
Both combined	All	Centromeric	24_en_7.89	225, 216	0.515	0.469–0.562	0.52
Both combined	All	Centromeric	13_en_16.75	119, 127	0.489	0.427–0.551	0.73
Both combined	All	Centromeric	26_st_0.2	47, 57	0.457	0.363–0.553	0.38
Female	F1	Distal	1_st_0.48	112, 105	0.521	0.455–0.587	0.53
Female	F1	Distal	1A_st_0.38	98, 119	0.457	0.391–0.523	0.20
Female	F1	Distal	2_en_155.77	113, 104	0.526	0.459–0.592	0.45
Female	F1	Distal	3_en_111.84	50, 45	0.531	0.431–0.630	0.54
Female	F1	Distal	4_en_69.2	115, 102	0.535	0.469–0.601	0.30
Female	F1	Distal	4A_st_0.45	84, 103	0.454	0.384–0.526	0.21
Female	F1	Distal	5_en_62.17	110, 107	0.512	0.446–0.578	0.72
Female	F1	Distal	6_en_35.99	101, 94	0.523	0.453–0.592	0.52
Female	F1	Distal	7_en_39.18	117, 98	0.549	0.483–0.615	0.15
Female	F1	Distal	8_en_27.41	103, 114	0.480	0.414–0.546	0.55
Female	F1	Distal	9_en_26.74	102, 115	0.475	0.409–0.542	0.47
Female	F1	Distal	10_en_20.56	120, 97	0.558	0.492–0.623	0.087
Female	F1	Distal	11_st_0.14	97, 120	0.452	0.387–0.519	0.16
Female	F1	Distal	14_en_15.44	112, 105	0.521	0.455–0.587	0.53
Female	F1	Distal	15_st_0.88	112, 105	0.521	0.455–0.587	0.53
Female	F1	Distal	19_en_11.22	89, 124	0.423	0.358–0.490	0.025
Female	F1	Distal	20_en_15.24	116, 101	0.540	0.473–0.605	0.24
Female	F1	Distal	21_en_5.8	108, 109	0.503	0.437–0.569	0.93
Female	F1	Distal	22_st_0.13	102, 115	0.475	0.409–0.542	0.47
Female	F1	Distal	23_en_6.19	115, 102	0.535	0.469–0.601	0.30
Female	F1	Distal	24_st_0.41	101, 116	0.471	0.405–0.537	0.39
Female	F1	Distal	25_en_1.22	55, 65	0.463	0.376–0.553	0.42
Female	F1	Distal	26_en_4.78	104, 112	0.487	0.420–0.553	0.69
Female	F1	Distal	13_st_0.37	90, 102	0.474	0.404–0.545	0.47
Female	BC1	Distal	1_st_0.48	116, 118	0.501	0.437–0.565	0.98
Female	BC1	Distal	1A_st_0.38	131, 144	0.482	0.423–0.541	0.54
Female	BC1	Distal	6_en_35.99	133, 125	0.521	0.460–0.581	0.51
Female	BC1	Distal	9_en_26.74	125, 137	0.482	0.422–0.543	0.57
Female	BC1	Distal	10_en_20.56	123, 108	0.538	0.473–0.601	0.25
Female	BC1	Distal	11_st_0.14	146, 158	0.485	0.429–0.542	0.61
Female	BC1	Distal	14_en_15.44	81, 88	0.484	0.410–0.560	0.69
Female	BC1	Distal	15_st_0.88	118, 101	0.544	0.478–0.609	0.19
Female	BC1	Distal	19_en_11.22	22, 32	0.412	0.287–0.546	0.20
Female	BC1	Distal	20_en_15.24	79, 56	0.590	0.506–0.671	0.037
Female	BC1	Distal	23_en_6.19	105, 125	0.462	0.398–0.526	0.25
Female	BC1	Distal	24_st_0.41	83, 66	0.562	0.482–0.640	0.13
Female	BC1	Distal	26_en_4.78	118, 116	0.509	0.446–0.573	0.77

Female	BC1	Distal	4_en_69.2	122, 121	0.507	0.445–0.570	0.82
Female	BC1	Distal	4A_st_0.45	133, 98	0.581	0.517–0.643	0.014
Female	BC1	Distal	8_en_27.41	166, 170	0.499	0.446–0.553	0.98
Female	BC1	Distal	22_st_0.13	74, 79	0.489	0.410–0.568	0.78
Female	BC1	Distal	25_en_1.22	72, 85	0.464	0.387–0.542	0.36
Female	BC1	Distal	2_en_155.77	158, 143	0.530	0.474–0.586	0.30
Female	BC1	Distal	3_en_111.84	19, 30	0.393	0.264–0.532	0.14
Female	BC1	Distal	5_en_62.17	141, 164	0.467	0.412–0.524	0.26
Female	BC1	Distal	7_en_39.18	40, 31	0.568	0.452–0.679	0.25
Female	BC1	Distal	13_st_0.37	104, 126	0.457	0.394–0.522	0.20
Female	BC1	Distal	21_en_5.8	23, 15	0.610	0.452–0.754	0.18
Female	All	Distal	1_st_0.48	228, 223	0.511	0.465–0.557	0.65
Female	All	Distal	1A_st_0.38	229, 263	0.471	0.427–0.515	0.19
Female	All	Distal	2_en_155.77	271, 247	0.528	0.485–0.571	0.20
Female	All	Distal	3_en_111.84	69, 75	0.484	0.403–0.566	0.71
Female	All	Distal	4_en_69.2	237, 223	0.520	0.475–0.566	0.38
Female	All	Distal	4A_st_0.45	217, 201	0.524	0.476–0.572	0.32
Female	All	Distal	5_en_62.17	251, 271	0.486	0.443–0.529	0.52
Female	All	Distal	6_en_35.99	234, 219	0.522	0.476–0.567	0.36
Female	All	Distal	7_en_39.18	157, 129	0.554	0.496–0.611	0.068
Female	All	Distal	8_en_27.41	269, 284	0.492	0.450–0.533	0.69
Female	All	Distal	9_en_26.74	227, 252	0.479	0.434–0.524	0.36
Female	All	Distal	10_en_20.56	243, 205	0.548	0.501–0.593	0.044
Female	All	Distal	11_st_0.14	243, 278	0.472	0.429–0.514	0.19
Female	All	Distal	14_en_15.44	193, 193	0.505	0.455–0.555	0.84
Female	All	Distal	15_st_0.88	230, 206	0.533	0.486–0.579	0.17
Female	All	Distal	19_en_11.22	111, 156	0.421	0.362–0.481	0.010
Female	All	Distal	20_en_15.24	195, 157	0.559	0.507–0.610	0.027
Female	All	Distal	21_en_5.8	131, 124	0.519	0.458–0.580	0.55
Female	All	Distal	22_st_0.13	176, 194	0.481	0.430–0.532	0.46
Female	All	Distal	23_en_6.19	220, 227	0.497	0.451–0.544	0.91
Female	All	Distal	24_st_0.41	184, 182	0.508	0.457–0.559	0.76
Female	All	Distal	25_en_1.22	127, 150	0.464	0.405–0.522	0.23
Female	All	Distal	26_en_4.78	222, 228	0.498	0.452–0.545	0.95
Female	All	Distal	13_st_0.37	194, 228	0.465	0.418–0.513	0.15
Male	F1	Distal	1_st_0.48	104, 120	0.469	0.405–0.535	0.36
Male	F1	Distal	1A_st_0.38	102, 122	0.460	0.396–0.526	0.24
Male	F1	Distal	2_en_155.77	116, 108	0.523	0.458–0.588	0.49
Male	F1	Distal	4_en_69.2	114, 110	0.514	0.449–0.579	0.67
Male	F1	Distal	4A_st_0.45	89, 93	0.494	0.422–0.566	0.87
Male	F1	Distal	5_en_62.17	113, 111	0.510	0.444–0.575	0.77
Male	F1	Distal	6_en_35.99	102, 122	0.460	0.396–0.526	0.24
Male	F1	Distal	7_en_39.18	123, 101	0.554	0.489–0.618	0.10
Male	F1	Distal	8_en_27.41	106, 118	0.478	0.413–0.544	0.52
Male	F1	Distal	9_en_26.74	112, 112	0.505	0.440–0.570	0.88
Male	F1	Distal	10_en_20.56	102, 122	0.460	0.396–0.526	0.24
Male	F1	Distal	11_st_0.14	106, 118	0.478	0.413–0.544	0.52
Male	F1	Distal	13_st_0.37	103, 121	0.465	0.400–0.530	0.29
Male	F1	Distal	14_en_15.44	96, 99	0.497	0.428–0.567	0.94
Male	F1	Distal	15_st_0.88	97, 127	0.438	0.374–0.504	0.065
Male	F1	Distal	19_en_11.22	105, 116	0.480	0.415–0.546	0.56
Male	F1	Distal	20_en_15.24	121, 103	0.545	0.480–0.610	0.18
Male	F1	Distal	21_en_5.8	66, 63	0.517	0.431–0.602	0.70
Male	F1	Distal	22_st_0.13	115, 106	0.526	0.460–0.591	0.45
Male	F1	Distal	23_en_6.19	98, 126	0.443	0.378–0.508	0.087
Male	F1	Distal	24_st_0.41	116, 108	0.523	0.458–0.588	0.49
Male	F1	Distal	26_en_4.78	101, 122	0.458	0.393–0.524	0.21
Male	F1	Distal	Z_en_72.81	115, 109	0.519	0.453–0.583	0.58

Male	F1	Distal	3_en_111.84	74, 58	0.566	0.481–0.648	0.13
Male	F1	Distal	28_en_4.93	40, 26	0.611	0.491–0.722	0.073
Male	F1	Distal	18_en_10.64	37, 31	0.549	0.431–0.664	0.42
Male	F1	Distal	25_en_1.22	58, 47	0.557	0.462–0.650	0.24
Male	BC1	Distal	2_en_155.77	159, 172	0.486	0.432–0.539	0.60
Male	BC1	Distal	4_en_69.2	80, 76	0.518	0.440–0.596	0.65
Male	BC1	Distal	6_en_35.99	87, 76	0.539	0.462–0.614	0.32
Male	BC1	Distal	8_en_27.41	167, 167	0.505	0.452–0.559	0.85
Male	BC1	Distal	15_st_0.88	104, 108	0.496	0.429–0.563	0.90
Male	BC1	Distal	20_en_15.24	119, 88	0.580	0.512–0.646	0.022
Male	BC1	Distal	22_st_0.13	104, 104	0.505	0.437–0.573	0.88
Male	BC1	Distal	23_en_6.19	58, 92	0.392	0.316–0.471	0.0086
Male	BC1	Distal	25_en_1.22	106, 98	0.525	0.456–0.593	0.48
Male	BC1	Distal	9_en_26.74	85, 98	0.470	0.398–0.542	0.41
Male	BC1	Distal	21_en_5.8	18, 30	0.380	0.251–0.521	0.10
Male	BC1	Distal	24_st_0.41	95, 90	0.519	0.447–0.590	0.61
Male	BC1	Distal	1_st_0.48	42, 36	0.544	0.433–0.651	0.44
Male	BC1	Distal	1A_st_0.38	106, 105	0.508	0.440–0.575	0.83
Male	BC1	Distal	5_en_62.17	69, 80	0.468	0.389–0.548	0.44
Male	BC1	Distal	10_en_20.56	82, 88	0.488	0.413–0.562	0.74
Male	BC1	Distal	11_st_0.14	106, 100	0.520	0.452–0.587	0.57
Male	BC1	Distal	26_en_4.78	75, 75	0.505	0.426–0.585	0.90
Male	BC1	Distal	4A_st_0.45	107, 106	0.508	0.441–0.574	0.83
Male	BC1	Distal	13_st_0.37	77, 84	0.483	0.407–0.560	0.67
Male	BC1	Distal	14_en_15.44	59, 81	0.426	0.346–0.509	0.083
Male	BC1	Distal	7_en_39.18	36, 33	0.527	0.410–0.642	0.66
Male	BC1	Distal	3_en_111.84	32, 20	0.620	0.485–0.744	0.085
Male	BC1	Distal	Z_en_72.81	17, 8	0.684	0.491–0.842	0.071
Male	BC1	Distal	19_en_11.22	82, 103	0.448	0.378–0.520	0.16
Male	All	Distal	1_st_0.48	146, 156	0.489	0.432–0.545	0.69
Male	All	Distal	1A_st_0.38	208, 227	0.483	0.437–0.530	0.49
Male	All	Distal	2_en_155.77	275, 280	0.501	0.459–0.542	0.98
Male	All	Distal	4_en_69.2	194, 186	0.516	0.465–0.566	0.54
Male	All	Distal	4A_st_0.45	196, 199	0.501	0.452–0.551	0.96
Male	All	Distal	5_en_62.17	182, 191	0.493	0.443–0.544	0.79
Male	All	Distal	6_en_35.99	189, 198	0.494	0.444–0.543	0.80
Male	All	Distal	7_en_39.18	159, 134	0.548	0.491–0.604	0.10
Male	All	Distal	8_en_27.41	273, 285	0.494	0.453–0.536	0.79
Male	All	Distal	9_en_26.74	197, 210	0.489	0.441–0.538	0.66
Male	All	Distal	10_en_20.56	184, 210	0.472	0.423–0.522	0.27
Male	All	Distal	11_st_0.14	212, 218	0.498	0.451–0.545	0.94
Male	All	Distal	13_st_0.37	180, 205	0.473	0.423–0.523	0.28
Male	All	Distal	14_en_15.44	155, 180	0.468	0.415–0.521	0.24
Male	All	Distal	15_st_0.88	201, 235	0.466	0.420–0.513	0.16
Male	All	Distal	19_en_11.22	187, 219	0.466	0.417–0.514	0.17
Male	All	Distal	20_en_15.24	240, 191	0.562	0.515–0.608	0.010
Male	All	Distal	21_en_5.8	84, 93	0.480	0.407–0.553	0.59
Male	All	Distal	22_st_0.13	219, 210	0.516	0.468–0.563	0.52
Male	All	Distal	23_en_6.19	156, 218	0.422	0.373–0.473	0.0028
Male	All	Distal	24_st_0.41	211, 198	0.521	0.473–0.569	0.39
Male	All	Distal	26_en_4.78	176, 197	0.477	0.427–0.528	0.37
Male	All	Distal	Z_en_72.81	132, 117	0.535	0.473–0.597	0.27
Male	All	Distal	3_en_111.84	106, 78	0.581	0.509–0.651	0.028
Male	All	Distal	28_en_4.93	40, 26	0.611	0.491–0.722	0.073
Male	All	Distal	18_en_10.64	37, 31	0.549	0.431–0.664	0.42
Male	All	Distal	25_en_1.22	164, 145	0.536	0.480–0.591	0.21
Both combined	F1	Distal	1_st_0.48	216, 225	0.495	0.448–0.542	0.83
Both combined	F1	Distal	1A_st_0.38	200, 241	0.459	0.412–0.505	0.083

Both combined	F1	Distal	2_en_155.77	229, 212	0.524	0.478–0.571	0.31
Both combined	F1	Distal	4_en_69.2	229, 212	0.524	0.478–0.571	0.31
Both combined	F1	Distal	4A_st_0.45	173, 196	0.474	0.423–0.525	0.32
Both combined	F1	Distal	5_en_62.17	223, 218	0.511	0.464–0.557	0.65
Both combined	F1	Distal	6_en_35.99	203, 216	0.490	0.442–0.537	0.67
Both combined	F1	Distal	7_en_39.18	240, 199	0.552	0.505–0.598	0.030
Both combined	F1	Distal	8_en_27.41	209, 232	0.479	0.433–0.526	0.38
Both combined	F1	Distal	9_en_26.74	214, 227	0.490	0.444–0.537	0.69
Both combined	F1	Distal	10_en_20.56	222, 219	0.509	0.462–0.555	0.72
Both combined	F1	Distal	11_st_0.14	203, 238	0.465	0.419–0.512	0.15
Both combined	F1	Distal	13_st_0.37	193, 223	0.469	0.421–0.517	0.21
Both combined	F1	Distal	14_en_15.44	208, 204	0.510	0.462–0.558	0.68
Both combined	F1	Distal	15_st_0.88	209, 232	0.479	0.433–0.526	0.38
Both combined	F1	Distal	19_en_11.22	194, 240	0.452	0.406–0.499	0.047
Both combined	F1	Distal	20_en_15.24	237, 204	0.543	0.496–0.589	0.074
Both combined	F1	Distal	21_en_5.8	174, 172	0.508	0.455–0.560	0.76
Both combined	F1	Distal	22_st_0.13	217, 221	0.501	0.454–0.547	0.98
Both combined	F1	Distal	23_en_6.19	213, 228	0.488	0.442–0.535	0.62
Both combined	F1	Distal	24_st_0.41	217, 224	0.497	0.451–0.544	0.91
Both combined	F1	Distal	26_en_4.78	205, 234	0.472	0.426–0.519	0.24
Both combined	F1	Distal	Z_en_72.81	115, 109	0.519	0.453–0.583	0.58
Both combined	F1	Distal	3_en_111.84	124, 103	0.551	0.486–0.615	0.12
Both combined	F1	Distal	25_en_1.22	113, 112	0.507	0.442–0.572	0.82
Both combined	F1	Distal	28_en_4.93	40, 26	0.611	0.491–0.722	0.073
Both combined	F1	Distal	18_en_10.64	37, 31	0.549	0.431–0.664	0.42
Both combined	BC1	Distal	1_st_0.48	158, 154	0.512	0.456–0.567	0.68
Both combined	BC1	Distal	1A_st_0.38	237, 249	0.493	0.448–0.537	0.75
Both combined	BC1	Distal	6_en_35.99	220, 201	0.528	0.480–0.575	0.26
Both combined	BC1	Distal	9_en_26.74	210, 235	0.477	0.431–0.524	0.33
Both combined	BC1	Distal	10_en_20.56	205, 196	0.516	0.467–0.565	0.51
Both combined	BC1	Distal	11_st_0.14	252, 258	0.499	0.456–0.543	0.97
Both combined	BC1	Distal	14_en_15.44	140, 169	0.458	0.403–0.514	0.14
Both combined	BC1	Distal	15_st_0.88	222, 209	0.520	0.473–0.567	0.40
Both combined	BC1	Distal	19_en_11.22	104, 135	0.440	0.378–0.504	0.066
Both combined	BC1	Distal	20_en_15.24	198, 144	0.584	0.531–0.635	0.0020
Both combined	BC1	Distal	23_en_6.19	163, 217	0.434	0.385–0.484	0.010
Both combined	BC1	Distal	24_st_0.41	178, 156	0.538	0.484–0.591	0.16
Both combined	BC1	Distal	26_en_4.78	193, 191	0.508	0.458–0.558	0.76
Both combined	BC1	Distal	4_en_69.2	202, 197	0.511	0.462–0.560	0.65
Both combined	BC1	Distal	4A_st_0.45	240, 204	0.546	0.499–0.592	0.054
Both combined	BC1	Distal	8_en_27.41	333, 337	0.502	0.464–0.540	0.91
Both combined	BC1	Distal	22_st_0.13	178, 183	0.498	0.447–0.550	0.95
Both combined	BC1	Distal	25_en_1.22	178, 183	0.498	0.447–0.550	0.95
Both combined	BC1	Distal	2_en_155.77	317, 315	0.507	0.468–0.546	0.73
Both combined	BC1	Distal	3_en_111.84	51, 50	0.510	0.413–0.606	0.84
Both combined	BC1	Distal	5_en_62.17	210, 244	0.468	0.422–0.514	0.17
Both combined	BC1	Distal	7_en_39.18	76, 64	0.548	0.465–0.629	0.26
Both combined	BC1	Distal	13_st_0.37	181, 210	0.468	0.419–0.518	0.21
Both combined	BC1	Distal	21_en_5.8	41, 45	0.482	0.378–0.587	0.74
Both combined	BC1	Distal	Z_en_72.81	17, 8	0.684	0.491–0.842	0.071
Both combined	All	Distal	1_st_0.48	374, 379	0.502	0.466–0.537	0.92
Both combined	All	Distal	1A_st_0.38	437, 490	0.477	0.444–0.509	0.15
Both combined	All	Distal	2_en_155.77	546, 527	0.514	0.484–0.544	0.36
Both combined	All	Distal	4_en_69.2	431, 409	0.518	0.484–0.552	0.29
Both combined	All	Distal	4A_st_0.45	413, 400	0.513	0.479–0.547	0.45
Both combined	All	Distal	5_en_62.17	433, 462	0.489	0.456–0.522	0.51
Both combined	All	Distal	6_en_35.99	423, 417	0.509	0.475–0.542	0.61
Both combined	All	Distal	7_en_39.18	316, 263	0.551	0.510–0.591	0.014

Both combined	All	Distal	8_en_27.41	542, 569	0.493	0.464–0.522	0.64
Both combined	All	Distal	9_en_26.74	424, 462	0.484	0.451–0.517	0.33
Both combined	All	Distal	10_en_20.56	427, 415	0.512	0.479–0.546	0.48
Both combined	All	Distal	11_st_0.14	455, 496	0.484	0.452–0.515	0.31
Both combined	All	Distal	13_st_0.37	374, 433	0.469	0.434–0.503	0.075
Both combined	All	Distal	14_en_15.44	348, 373	0.488	0.451–0.524	0.51
Both combined	All	Distal	15_st_0.88	431, 441	0.499	0.466–0.533	0.97
Both combined	All	Distal	19_en_11.22	298, 375	0.448	0.411–0.486	0.0070
Both combined	All	Distal	20_en_15.24	435, 348	0.561	0.526–0.595	7×10^{-4}
Both combined	All	Distal	21_en_5.8	215, 217	0.503	0.456–0.550	0.91
Both combined	All	Distal	22_st_0.13	395, 404	0.500	0.465–0.534	0.98
Both combined	All	Distal	23_en_6.19	376, 445	0.463	0.429–0.497	0.035
Both combined	All	Distal	24_st_0.41	395, 380	0.515	0.480–0.550	0.41
Both combined	All	Distal	26_en_4.78	398, 425	0.489	0.455–0.523	0.52
Both combined	All	Distal	Z_en_72.81	132, 117	0.535	0.473–0.597	0.27
Both combined	All	Distal	3_en_111.84	175, 153	0.539	0.485–0.592	0.16
Both combined	All	Distal	25_en_1.22	291, 295	0.502	0.461–0.542	0.93
Both combined	All	Distal	28_en_4.93	40, 26	0.611	0.491–0.722	0.073
Both combined	All	Distal	18_en_10.64	37, 31	0.549	0.431–0.664	0.42

Table S7 | Transmission ratios in informative females of the BC1 generation against a background transmission rate of 0.495. Best linear unbiased estimates (BLUE) stem from a binomial GLMM with offset parameter 0.495 and female ID fitted as a random effect. CI = confidence interval.

**Chromosomal side**	***N* transmissions (A, T)**	**Ratio**	**BLUE**	**95% CI**	**P-value**	**Individual ID**	**P-value Individual ID**
Centromeric	2172, 2446	0.470	0.475	0.460–0.490	0.0012	0.0011	0.81
Distal	2432, 2436	0.500	0.503	0.487–0.519	0.69	0.0027	0.58
Both combined	4604, 4882	0.485	0.490	0.480–0.501	0.064	3×10^{-7}	1.00

Table S8 | Percentage of infertile eggs in the parental generation and in male and female hybrids in the F1 and BC1 generation. Because birds were bred in aviaries that contained either Timor males or females in the P generation or hybrid males or hybrid females in the F1 and BC1 generation, infertile eggs could be assigned to the sex that was homozygous or heterozygous for Timor alleles. Estimates were derived from a linear mixed-effects model, in which each egg was coded either as 0 = fertile or 1 = infertile. The clutch ID was fitted as a random effect.

Group	*N* eggs	*N* clutches	Infertility rate (%)	95% confidence interval
Australian female × Timor male	25	6	52.77	36.53–68.73
Australian female × F1 male	271	64	16.61	11.71–21.95
Australian female × BC1 male	554	131	17.26	13.66–21.1
Australian male × Timor female	11	3	21.07	-3.98–47.24
Australian male × F1 female	232	58	5.34	0.31–10.95
Australian male × BC1 female	476	131	5.92	2.16–9.72

References

BirdLife International and Handbook of the Birds of the World (2016a) *Taeniopygia castanotis*. The IUCN Red List of Threatened Species. Version 2017-3.

BirdLife International and Handbook of the Birds of the World (2016b) *Taeniopygia guttata*. The IUCN Red List of Threatened Species. Version 2017-3.

Knief U, Forstmeier W (2016) Mapping centromeres of microchromosomes in the zebra finch (*Taeniopygia guttata*) using half-tetrad analysis. *Chromosoma* **125**, 757–768.

Vazquez AI, Bates DM, Rosa GJM, Gianola D, Weigel KA (2010) Technical note: An R package for fitting generalized linear mixed models in animal breeding. *J Anim Sci* **88**, 497–504.

Warren WC, Clayton DF, Ellegren H, *et al*. (2010) The genome of a songbird. *Nature* **464**, 757–762.
